# Cell-specific image-guided transcriptomics identifies complex injuries caused by ischemic acute kidney injury in mice

**DOI:** 10.1038/s42003-019-0571-7

**Published:** 2019-09-02

**Authors:** Tomoaki Miyazaki, Sina A. Gharib, Yun-Wei A. Hsu, Katherine Xu, Pavlo Khodakivskyi, Akio Kobayashi, Jason Paragas, Alexander D. Klose, Kevin P. Francis, Elena Dubikovskaya, Patrick S. Page-McCaw, Jonathan Barasch, Neal Paragas

**Affiliations:** 1 Division of Nephrology, Department of Medicine, University of Washington, Seattle, WA 98195 USA; 2 Division of Nephrology, Department of Medicine, Showa University, Yokohama, Japan; 3 Computational Medicine Core, Center for Lung Biology, University of Washington, Seattle, WA 98195 USA; 4 Renal Division, Department of Medicine, Columbia University, New York, NY 10027 USA; 5 Institute of Chemical Sciences and Engineering, Swiss Federal Institute of Technology of Lausanne (EPFL), Lausanne, Switzerland; 6 Lawrence Livermore Labs, Livermore, CA 94550 USA; 7 InVivo Analytics, Inc., New York, NY 10023 USA; 8 PerkinElmer, Inc., Hopkinton, MA 01748 USA; 9 Division of Nephrology, Department of Medicine, Vanderbilt University Medical Center, Nashville, TN 37232 USA

**Keywords:** Bioluminescence imaging, Gene expression analysis

## Abstract

The kidney’s inherent complexity has made identifying cell-specific pathways challenging, particularly when temporally associating them with the dynamic pathophysiology of acute kidney injury (AKI). Here, we combine renal cell-specific luciferase reporter mice using a chemoselective luciferin to guide the acquisition of cell-specific transcriptional changes in C57BL/6 background mice. Hydrogen peroxide generation, a common mechanism of tissue damage, was tracked using a peroxy-caged-luciferin to identify optimum time points for immunoprecipitation of labeled ribosomes for RNA-sequencing. Together, these tools revealed a profound impact of AKI on mitochondrial pathways in the collecting duct. In fact, targeting the mitochondria with an antioxidant, ameliorated not only hydrogen peroxide generation, but also significantly reduced oxidative stress and the expression of the AKI biomarker, LCN2. This integrative approach of coupling physiological imaging with transcriptomics and drug testing revealed how the collecting duct responds to AKI and opens new venues for cell-specific predictive monitoring and treatment.

## Abstract (web summary)

Miyazaki, Gharib, Hsu et al. use a combined cell-specific luciferase reporter system and chemo-selective substrate to identify mouse tissues at risk of renal injury. The platform can be used to identify cell-specific pathophysiological events and transcriptional changes, finding potential therapeutic targets.

## Introduction

In highly heterogenous organs such as the kidney, associating pathophysiological changes such as generation of reactive oxygen species (ROS) with transcriptional signals at the cell-type level are challenging, if not impossible. Furthermore, monitoring these pathophysiological intercellular changes non-invasively and longitudinally has not been shown in the kidney. Here we present a coupled cell-specific image-guided transcriptomics approach to visualize ROS generation and associate it with gene expression changes in the injured kidney.

In vivo methods for optically monitoring cell-specific or temporal changes in reactive oxygen species (ROS) have been previously described in transplanted tissues^1^; however, none with a cell-specific approach within the heterogeneous structures of the kidney. Standard methods for quantifying oxidative stress has required serial sacrifice and measurement of lipid peroxidation or byproducts such as malondialdehyde (MDA)^2^. In addition, DNA/RNA damage, hydrogen peroxide (H_2_O_2_) and nitric oxide levels, as well as changes in antioxidants such as glutathione and catalase, have been measured ex vivo to assess oxidative stress. However, each of these methods is static and provides only cross-sectional assessments of oxidative stress without cell specificity.

The unmet need to identify and map damage to different types of epithelia within the nephron stems from our inadequate understanding of the diverse causes and cellular responses to acute changes in kidney function, currently lumped together under the umbrella term of acute kidney injury (AKI)^3–5^. The kidney is derived from the ureteric bud and the metanephrogenic mesenchyme; these two progenitor cell populations differentiate into more than 26 different cell types in the adult kidney^6^.

One common and essential mechanism for many types of AKI is unhindered activation of oxidative stress programs. Oxidative stress is generally a result of the cessation and subsequent reperfusion of renal blood flow leading to the generation of ROS. Excessive ROS can cause lipid peroxidation, DNA damage, inactivation of antioxidants, and cytoskeleton reorganization, which leads to inflammation, fibrosis and eventually the irreversible condition of end stage renal disease (ESRD)^7^. Oxidative stress can be induced by ischemia-reperfusion injury (IRI), shock, sepsis, surgery and the administration of imaging contrast or other nephrotoxic agents^8,9^. However, these processes have eluded targeted analysis because of the paucity of effective methods to visualize the sites of damage.

Here, we report the generation of a group of cell-specific transgenic luciferase reporter mice to longitudinally monitor H_2_O_2_ generation after kidney IRI. We quantified oxidative stress in a non-invasive, tissue-specific manner using bioluminescence as a readout to identify the optimal time point for cell-specific transcriptional analyses. We found that ischemia-reperfusion induced oxidative stress generation in different segments of the kidney but with different timing and dose sensitivity, a finding that sheds light on a long-standing debate concerning the relative responses of different kidney segments. Biomarkers of oxidative stress damage were significantly reduced following pretreatment with an antioxidant that moderated the ischemic injury in vivo. In sum, the ability to dynamically monitor cell-specific ROS generation and use it to pinpoint the optimum time for cell-specific transcriptional interrogation in animal models of human kidney disease provides a unique opportunity to understand the impact of different forms of AKI on the different cell types of the kidney. Most importantly, this coupled approach that exploits a cell-specific luciferase reporter with a chemoselective luciferin to guide time- and cell-specific RNA-isolation and transcriptional analysis can be applied to many other mouse models of human disease.

## Results

### Non-invasive cell-specific imaging of kidney cells

**Fig. 1:**
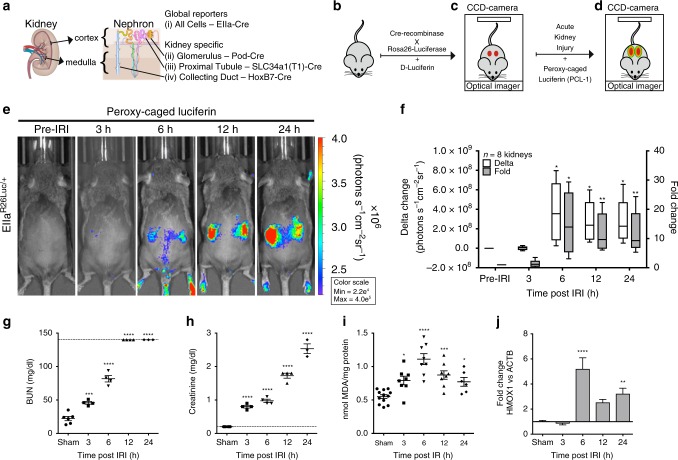
Non-invasive monitoring of oxidative stress using nephron segment specific luciferase reporter mice. **a** Global luciferase reporter animal, *EIIa*^*R26Luc/+*^. Glomerular luciferase reporter animal, *Pod*^*R26Luc/+*^. Proximal tubule (S1/S2) reporter animal, *Slc34a1*^*R26Luc/+*^. Collecting duct luciferase reporter animal, *HoxB7*^*R26Luc/+*^. Images created by Kate Sweeney. **b** Cre-mediated Rosa26-luciferase reporter animals were generated by breeding R26-luciferase reporter mice with tissue-specific Cre-recombinase mice. **c** Luciferase reporter activity was confirmed by injection of D-luciferin (150 mg/kg). **d** Luciferase reporter mice were challenged with IRI and ROS (H_2_O_2_) activity was non-invasively monitored with an optical imaging system which detected H_2_O_2_ activated caged-luciferin probe, PC-Luciferin. **e** *EIIa*^*R26Luc/+*^ mice were challenged with 30 min of bilateral IRI and monitored with PC-Luciferin over 24 h (*n* *=* 8; representative longitudinal image of one animal). **f** Delta and fold change of kidney PC-Luciferin (10 mg/kg) signal from ROIs drawn over kidneys with pre-IRI signal used as the denominator for the entire time course. **g**, **h** Blood parameters of C57BL/6 mice challenged with 30 min of bilateral IRI acquired with clinical blood analyzer. **g** Blood urea nitrogen (BUN) and **h** serum creatinine (sCr) significantly increased 3, 6, 12, and 24 h after IRI compared with sham animals. Line indicates the limits of detection of the BUN and sCr assay (140 mg/dl and 0.2 mg/dl, respectively; *n* *=* 6 mice for sham, *n* *=* 4 for 3, 6, and 12 h and *n* *=* 3 for 24 h after IRI). **i** Whole kidney levels of malondialdehyde (MDA), a naturally occurring product of lipid peroxidation, increased significantly 3–12 h after IRI compared with sham animals (*n* = 12 kidneys for sham, *n* = 8 kidneys for 3, 6, and 12 h and *n* *=* 6 for 24 h after IRI). **j** Matched qPCR shows Hmox1, involved in heme degradation, was significantly upregulated at 6 h. qPCR: *n* = 10 kidneys for sham, *n* = 8 kidneys for 3, 6, and 12 h after IRI, and *n* = 6 kidneys for 24 h after IRI. All data: *P < 0.05, **P < 0.01, ***P < 0.001, and ****P < 0.0001 vs. sham

In order to image ROS generation following kidney injury, we generated both global luciferase and tissue-specific reporters—the glomerulus (Podocin promoter), the proximal tubule (Slc34a1 promoter), and the collecting duct (HoxB7 promoter) (Fig. 1a). Activation of the firefly *luciferase* (*luc*) gene required Cre-mediated recombination of a loxP-flanked STOP fragment intercalated between the *luc* sequence and the *Gt(ROSA)26Sor* promoter^10^ (Fig. 1b) by the following Cre-recombinase mice: *EIIa-Cre*, with Cre-recombinase expression in every cell-type; Pod-Cre, with Cre-recombinase expression in podocin-positive glomerular cells under the control of the human *NPHS2* gene, to make *Pod*^*R26Luc/+*^; Slc34a1-Cre, with Cre-recombinase expression in S1 and S2 segments of the proximal tubule cells, to make to make *Slc34a1*^*R26Luc/+*^; and HoxB7-Cre, with Cre-recombinase expression in mesonephric duct and its derivatives, which includes the collecting duct and ureteral epithelia, to make *HoxB7*^*R26Luc/+*^ (Fig. 1a–c and Supplementary Table 1)^11–14^. Bioluminescence emanating from these specific reporter cells was captured in vivo by an optical imaging system (IVIS Spectrum, PerkinElmer) and interpreted using Living-Image (PerkinElmer) and InVivoAX (InVivo Analytics) software. Recombination was confirmed in *EIIa*^*R26Luc/+*^, *Pod*^*R26Luc/+*^, *Slc34a1*^*R26Luc/+*^, and *HoxB7*^*R26Luc/+*^ luciferase reporter mice by imaging luciferase activity with a saturating dose of D-Luciferin (150 mg/kg: Supplementary Figs. 1–4, Supplementary Movies 1–3)^15^. Kidney-specific luciferase reporter activity was confirmed using bioluminescent tomographic reconstruction and alignment to the digital mouse atlas (InVivoPLOT)^16^. Luciferase reporter mice illuminated the expected expression domains in the kidney including the entire kidney (*EIIa*-promoter) (Supplementary Fig. 4a), and specific segments of the nephron (*HoxB7*, *Slc34a1*, and *Podocin* promoters; Supplementary Figs. 1–3) using D-luciferin (150 mg/kg).

### Longitudinal progression of oxidative stress in injured kidney

To simulate AKI in a manner consistent with clinical settings, we challenged conditional luciferase reporter mice with bilateral IRI to induce ROS^17^. Either 30 min of bilateral IRI or sham surgery was used in order to reproduce previous studies where AKI caused significant oxidative stress related injury, previously measured ex vivo^18^. A modified version of a H_2_O_2_ reporter caged-luciferin, called peroxy-caged luciferin (PC-Luciferin), was used to quantify Cre dependent, cell-specific, H_2_O_2_ generation (Supplementary Fig. 5)^1^. Chemical caging of firefly luciferin with alkyl group (selectively cleavable) prevents production of light from luciferin/luciferase reaction. As the result, no light is produced in the absence of a cleaving agent, H_2_O_2_. PC-Luciferin, unlike free D-Luciferin, is incapable of undergoing the luciferin/luciferase reaction unless first hydrolyzed by H_2_O_2_; hence, PC-Luciferin selectively detects H_2_O_2_ production in a concentration-dependent manner^1^.

To longitudinally and repetitively monitor ROS in vivo with a non-invasive and cell-specific method, we repetitively collected whole body optical images from the same reporter mouse 3, 6, 12, and 24 h after IRI using PC-luciferin (10 mg/kg) to map spatiotemporal H_2_O_2_ expression. The global luciferase reporter mouse, *EIIa*^*R26Luc/+*^, revealed a significant increase in H_2_O_2_ reporter activity in both kidneys 6 h (fold change: *P* = 0.0175) after IRI challenge (Fig. 1e, f). The *EIIa*^*R26Luc/+*^ luciferase-luciferin signal was not significantly modulated by the injury (Supplementary Fig. 4b, c); therefore, in this model the caged-luciferin activity is not impaired by changes in luciferase enzymatic activity.

In order to determine whether the time course of oxidative stress was associated with classical markers of AKI we obtained sequential in vivo measurements of oxidative stress with concomitant measurements of tissue markers of lipid peroxidation (LPOs) at 3, 6, 12, and 24 h after IRI. Blood parameters were analyzed using a handheld clinical analyzer (Fig. 1g, h and Supplementary Table 2). To calibrate our in vivo measurements against established markers of IRI-AKI, excised kidneys were bisected and one half was processed for MDA (Fig. 1i and Supplementary Table 3) while total RNA was extracted from the other. Consistent with previous studies, IRI of the kidney resulted in significant increase in blood urea nitrogen (BUN: *P* < 0.001), serum creatinine (sCr: *P* < 0.0001), and LPOs (*P* < 0.05) over the 24 h period post injury (Fig. 1g–i)^18^. In vivo measures of *EIIa*^*R26Luc/+*^ PC-luciferin activity were in agreement with MDA levels, at 6 h after IRI (Fig. 1e, f, i). Hmox1, a gene induced by oxidative damage and a potential antioxidant, trended with MDA levels and the caged PC-Luciferin yield, indicating that peak oxidative stress occurred at 6 h in this model^19,20^. In sum, we demonstrated consistent associations between PC-Luciferin and classical markers of oxidation.

### Collecting duct is a dominant source of ROS following AKI

**Fig. 2:**
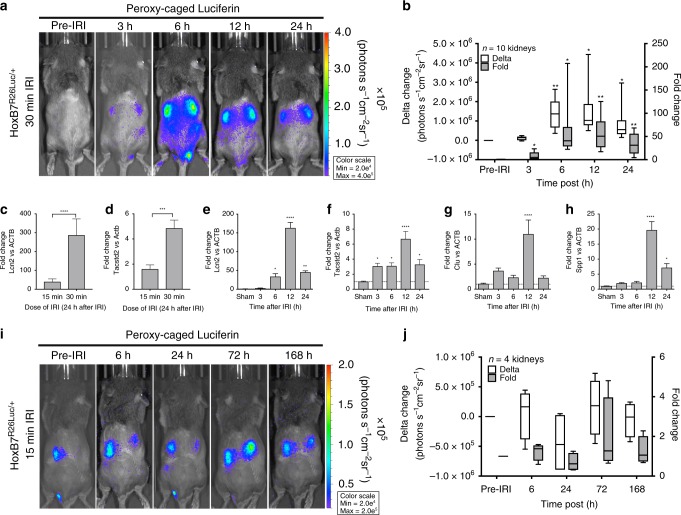
Collecting duct-specific H_2_O_2_ generation requires high dose of IRI. Intracellular H_2_O_2_ flux is reported by the production of light from the enzymatic reaction of luciferase and D-Luciferin the end product from the enzymatic reaction of PC-luciferin with H_2_O_2_. **a** Longitudinal time course of collecting duct luciferase reporter (*HoxB7*^*R26Luc/+*^) mouse (representative) with images acquired pre-IRI, and 3, 6, 12, and 24 h after IRI. **b** PC-Luciferin signal fold and absolute signal change (photons s^−1^ cm^−2^ sr^−1^) compared with pre-IRI levels from ROIs drawn over kidneys (*n* = 10). **c**, **d** Medullary kidney injury marker, Lcn2, was measured by real-time PCR from whole kidney total RNA, and **d** Tacstd2, a novel collecting duct injury marker increased expression after 15 min and 30 min of IRI in a dose-responsive fashion. **e** *Lcn2* and was significantly upregulated 6–24 h after reperfusion. **f** *Tacstd2* was significantly upregulated 3–24 h after IRI. **g**, **h** Additional distal injury markers, *Clu* and *Spp1*, showed significant elevations 12 h post injury. qPCR: *n* = 10 kidneys for sham, *n* = 8 kidneys for 3, 6, and 12 h after IRI, and *n* = 6 kidneys for 24 h after IRI. **i**, **j** Fifteen minutes of IRI does not induce a significant change in H_2_O_2_ activity. All data: *P < 0.05, **P < 0.01, ***P < 0.001, and ****P < 0.0001 vs. sham

An expression pattern similar to *EIIa*^*R26Luc/+*^ PC-luciferin (global) activity was found when *HoxB7*^*R26Luc/+*^ PC-luciferin (collecting duct) reporter mouse was challenged with IRI for 30 min (Fig. 2a, b). *HoxB7*^*R26Luc/+*^ showed a robust total change in photon emission (>10^6^ photons s^−1^ cm^−2^ sr^−1^) from baseline and a 61.2 ± 17.7 mean fold change (*P* = 0.0249) at the 6 h time point. Lcn2 (Fig. 2c), a medullary kidney injury biomarker and iron scavenger^15^, mirrored the ROS activity at this time point by being significantly elevated at 6 h (287-fold: *P* = 0.0114), which was consistent with our previous findings^15^. A new marker of kidney stress derived from the kidney medulla, Tacstd2^21^, was also significantly elevated (*P* *<* 0.05) in a dose-dependent manner (Fig. 2d). A time course of expression (Fig. 2e–f and Supplementary Table 4) confirmed a rapid genetic response to oxidative damage. Inflammatory cytokines Il1a, Il1b, and Il6, known to be induced by TNFa^22^ and hypoxia^23^ in ischemic renal injury, also peaked early. Egf, a candidate kidney injury biomarker, was significantly downregulated (*P* < 0.05) as observed clinically in patients with AKI and CKD. With a lower dose of IRI (15 min), collecting duct Lcn2 was activated only 39.9-fold, and ROS generation was restricted without a significant change in H_2_O_2_ reporter activity (Fig. 2i, j) or in LPOs generation^17^. These data imply that the collecting duct has a robust system of ROS scavenging, but these cells are still activated at lower doses of injury.

### Differential ROS generation in the nephron

**Fig. 3:**
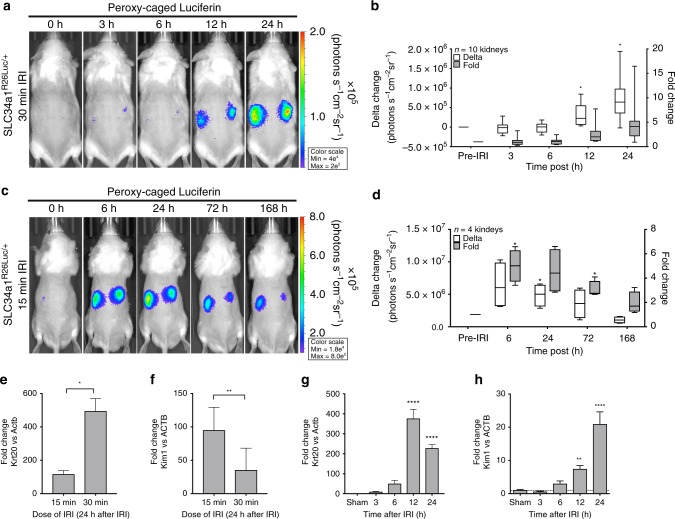
Proximal tubule-specific H_2_O_2_ generation requires low dose of IRI. **a**, **b** The proximal tubule luciferase reporter (*Slc34a1*^R26Luc/+^) significantly increased in absolute signal change (photons s^−1^ cm^−2^ sr^−1^) compared with pre-IRI levels from ROIs drawn over kidneys 12 and 24 h after 30 min of IRI. **c**, **d** 15 min of IRI induces a significant change in H_2_O_2_ activity beginning at 6 h after IRI and resolving after 168 h. **e** Krt20, a mixed proximal and intercalated cell marker, was significantly upregulated in after 15 and 30 min IRI in a dose-dependent manner, confirming a graded cortical injury. **f** However, pure proximal tubule injury marker, Kim1, expression was higher at a lower (15 min) than a higher dose (30 min) of IRI. Indicating Krt20 expression biased by activation collecting duct cell population. With 30 min of IRI, both **g** Krt20 **h** Kim1 significantly increased 12-24 h post ischemia, mirroring the H_2_O_2_ reporter activity in the proximal tubule. qPCR: *n* = 10 kidneys for sham, *n* = 8 kidneys for 3, 6, and 12 h after IRI, and *n* = 6 kidneys for 24 h after IRI. All data: *P < 0.05, **P < 0.01, ***P < 0.001, and ****P < 0.0001 vs. sham

In comparison to the collecting ducts, proximal tubule *Slc34a1*-Cre H_2_O_2_ dependent bioluminescence was delayed, and only significantly (*P* < 0.05) visible at 12 and 24 h following 30 min of IRI. Nor did it correlate with peak LPO signal. Furthermore, the proximal nephron H_2_O_2_ bioluminescent signal was a log order of magnitude less than the collecting duct even at its peak (Fig. 3a, b). The *Pod*^*R26Luc/+*^ mice also produced significant (*P* < 0.05) ROS-induced luciferase signal in the glomerular compartment, however, at a much smaller delta change in photon emission and overall fold change (Supplementary Fig. 6a, b). These results were unexpected since the proximal tubule comprises the majority of kidney epithelia and baseline reporter activity was comparable in each reporter demonstrating that this result is specific to the peroxide reaction, not luciferase expression (Supplementary Figs. 1–3). The limited response of the glomeruli was consistent with histological evidence that glomerular epithelia have limited gene responsiveness and evident injury in AKI and consistent with the limited response of the proximal tubules^20,21^ after 30 min of IRI. In addition, baseline luciferase activity (D-luciferin), which we monitored immediately after each PC-luciferin imaging session, was not significantly changed from baseline, suggesting that the IRI procedure did not impair luciferase reporter activity (Supplementary Fig. 4). Perhaps the limited H_2_O_2_ reporter activity in the proximal tubule was due to antioxidant scavenging activities which rapidly clear the ROS burden. Alternatively and most likely, enhanced sensitivity of this segment to hypoxic stress might render the cells either quiescent or apoptotic. To test the latter possibility, we examined the kidney at a reduced ischemic dose of 15 min IRI, monitored for a period of 7 days to observe recovery, and found a more rapid increase in H_2_O_2_ reporter activity in the proximal tubule (Fig. 3c, d). Expression of the proximal tubule injury biomarker *Kim1* (Fig. 3f) was upregulated 95-fold at 15 min post IRI, while 30 min of IRI resulted in 35-fold increase, which mirrored the *Slc34a1*-Luciferase H_2_O_2_ reporter findings of 4.6 × 10^6^ change in photons versus 7.2 × 10^5^ change at 24 h, respectively. A time course of proximal tubule kidney injury after 30 min of IRI with biomarkers such as Krt20 and Kim1^21,24^ demonstrated significant (*P* < 0.05) induction at 12 h after injury, consistent with the time course of expression of *Slc34a1*-Cre dependent luciferase (*Slc34a1*^*R26Luc/+*^) in the proximal tubule (Fig. 3b, g, h and Supplementary Table 4). The gene expression data indicate a significant (*P* < 0.05) activation of this compartment at 12 h after IRI, which mirrored H_2_O_2_ reporter activity, although substantially less than that generated by the collecting duct with 30 min of IRI.

In sum, by longitudinal monitoring of cell-specific oxidative stress in real-time, we demonstrated that the collecting duct is a particularly prominent and specific site of ROS generation. The response of the collecting duct differs in both timing and dose dependence to that of the proximal tubule. Our real-time ROS imaging data are consistent in timing with tissue-specific biomarkers (e.g., Lcn2, Krt20, and Kim1) and are in agreement with classical markers of cellular injury (sCr, BUN, and MDA).

### Transcriptional mapping of collecting duct after kidney IRI

**Fig. 4:**
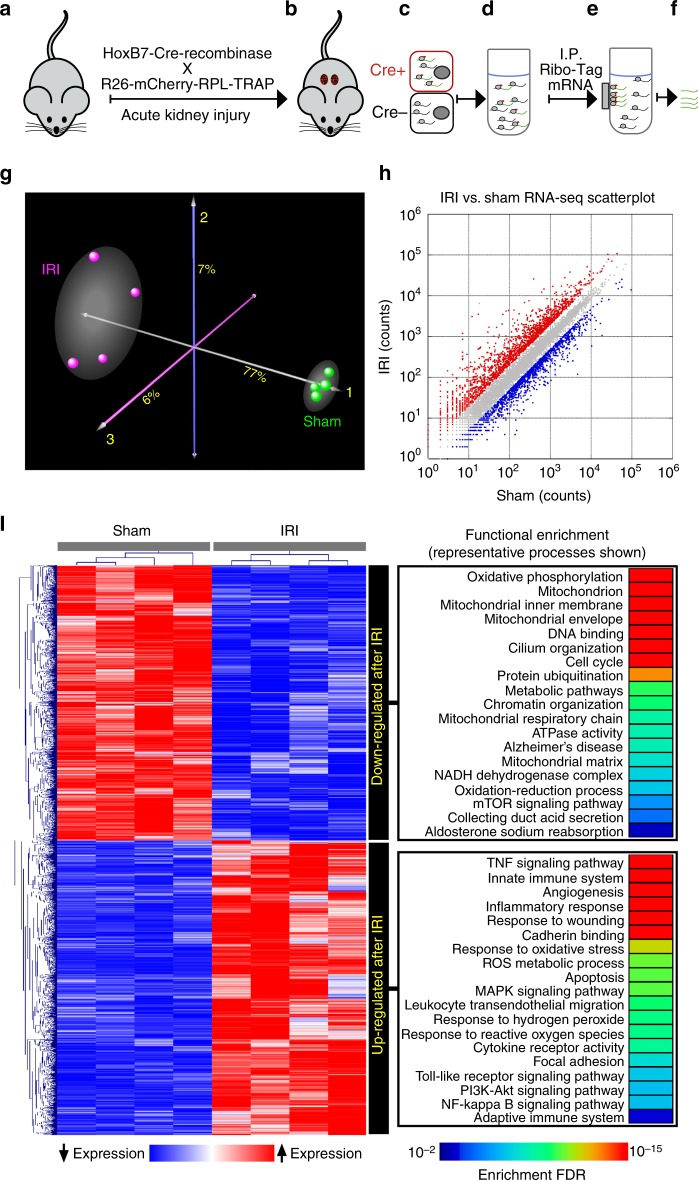
Transcriptomic (RNA-seq) and pathway analysis of HoxB7/Collecting Duct cells 9 h post IRI. **a** *HoxB7*-Cre mice were bred with *Gt(ROSA)26Sor-mCherry-Rpl10a* (RiboTRAP) mice to create collecting duct-specific RNA-isolation mouse that labels ribosomes for immunoprecipitation (IP). **b** Time point selected by cell-specific H_2_O_2_ generation (Figs. 1e and 2a), collecting duct RiboTRAP (*HoxB7*-mCherryRPL10a) kidneys were collected 9 h post IRI (6 h peak of H_2_O_2_ with time allotted for transcription; serum creatinine 1.1 ± 0.07 mg/Dl). At this time point **c** kidney-specific ribo-tagged kidneys were **d** homogenized, **e** magnetically immuno-precipitated, and **f** ribo-tagged transcripts isolated for RNA-sequencing. **g** Correspondence analysis depicting the global variability in the entire RNA-seq data. The IRI (*n* = 4) and sham (*n* = 4) experiments distinctly segregated, indicating transcriptome-wide effects, with the majority of the expression variability captured by the first component (77%). **h** Scatterplot of IRI vs sham RNA-seq counts shows a wide dynamic range of transcript abundance (~100,000 count difference). For each transcript, the average count from four biological replicates per conditions is shown. Differentially upregulated genes following IRI are colored in red whereas blue indicates downregulated genes (FDR < 0.01). Note the profound changes in gene expression in response to IRI. **i** Functional enrichment analysis of differentially expressed genes after hierarchical clustering. Up- and downregulated pathways (FDR < 0.01) were identified from multiple knowledgebases including KEGG, Reactome, and Gene Ontology. Note that IRI was associated with activation of multiple modules including those involved in inflammation/immunity, development, remodeling, and response to ROS. In addition, pathways mapping to mitochondrion such as oxidative phosphorylation and oxidoreductase activity were suppressed following IRI, suggesting widespread dysregulation of mitochondrial function

We were surprised by the prominent expression of oxidative stress by the collecting duct. In order to examine its mechanisms, we determined whether H_2_O_2_ imaging overlapped spatiotemporally with nascent gene expression (Fig. 4). Mice with collecting duct ribosome-tagged mRNA were generated by breeding Hoxb7-Cre with *Gt(ROSA)26Sor-mCherry-Rpl10a* (RiboTRAP) mice (Supplementary Fig. 7) and these *HoxB7*^*RPL/+*^ offspring were challenged with 30 min IRI (Fig. 4a, b). Kidneys were collected 9 h after injury which followed the peak of the H_2_O_2_ collecting duct signal. At this time point the injury induced transcriptional response^25^ is robust and serum creatinine (1.1 mg/dL) and tissue parameters (kidney injury biomarkers LCN2/KIM1) have confirmed initiation of injury. To confirm that the RiboTRAP system is specific for collecting duct we analyzed expression of *Aqp2* which is expressed specifically in the principal cells of the collecting duct. Aqp2 was enhanced by 18 ± 2.8-fold in pulldown of RiboTRAP mRNA compared with whole kidney RNA extract. RNA from four sham and four IRI HoxB7^RPL/+^ mice were isolated by magnetic immunoprecipitation (Fig. 4c–f) and sequenced with a HiSeq system (Illumina). We observed profound transcriptional changes in the collecting duct in response to ischemic injury with almost 8000 differentially up- and downregulated genes between IRI vs. sham HoxB7^+^ populations (FDR < 0.01) (Fig. 4g, h). Consistent with our previous observations, hypoxia inducible genes *Hspa1b* (4.4-fold) and *Hmox1* were upregulated (4.2-fold) along with the pro-inflammatory cytokines *Il1b* and *Il6* (12- and 190-fold). Interestingly, profibrotic genes were also modulated. *Rasal1* was downregulated 4.4-fold following IRI, consistent with the observation of RASAL1 hypermethylation leading to prolonged fibroblast activation and fibrogenesis after iAKI^26^. Tgfβ1 and Fgf2^27^ were upregulated by 4.0-fold and 9.5-fold, respectively, both of which can induce fibroblast activation in vivo^28^. In addition, S100A9, a cytoplasmic Ca binding protein, was also upregulated 35-fold in response to ischemia. Kidney injury genes Krt20 (proximal nephron), Clu, Spp1, Lcn2, and Tacstd2 (distal nephron) were upregulated 4.9-, 6.8-, 2.7-, 22-, and 2.7-fold, respectively. This sample of modulated genes confirms that the proximal nephron and the collecting ducts were activated by the injury.

To gain deeper insight into the transcriptional programs altered by IRI, we performed functional enrichment analysis on differentially expressed genes based on public databases, including KEGG, Reactome, and Gene Ontology. This analysis was applied separately to differentially up- and downregulated genes with FDR threshold of <0.01 to identify significantly enriched pathways (Fig. 4i). Activated modules following IRI included those involved in inflammation/immunity, development, apoptosis, and response to ROS. In addition, we observed downregulation of many mitochondrion-associated processes, implying that ischemic/hypoxic injury leads to a change in the functional state, from generating ATP to generating peroxide, in the collecting duct. In fact, 11 out of 13 detected members of the nuclear DNA expressed mitochondrial complex I (*Cx1*) genes (*NADH dehydrogenase (ubiquinone) alpha subcomplex* 1-12) were significantly downregulated by an average of 1.3-fold (FDR range: 4.5E−20 to 0.03). *Cx1* impairment and downregulation is known to cause H_2_O_2_ and ROS generation^29,30^.

In sum, our results show that leveraging the ROS-reporter animal uncovered the key pathways and kinetics of oxidative stress. Our results also distinguish generalized functional (sCr and BUN) and inflammatory responses (Hspa1b, Il1b) from ROS-associated responses (PC-Luciferin; Hmox1, Lcn2).

### Reduction of ROS cellular injury with an antioxidant

**Fig. 5:**
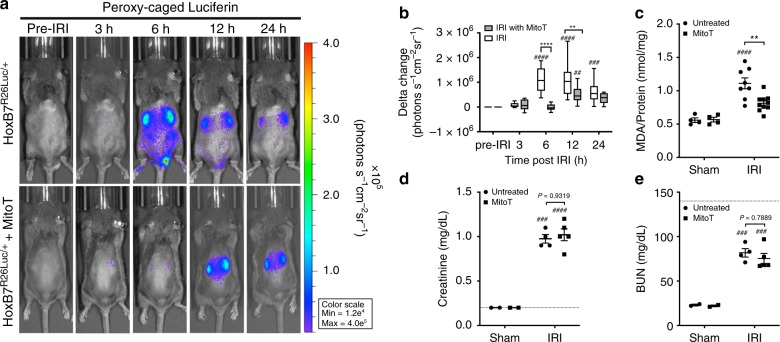
MitoTEMPO pretreatment inhibits kidney ROS generation. ROS generation in *HoxB7*^*R26Luc/+*^ mice with MitoT (10 mg/kg) treatment compared with vehicle control IRI animals was longitudinally monitored by an optical imager over a period of 24 h (representative mice) using the H_2_O_2_ sensitive luciferase reporter, PC-Luciferin. **a** *HoxB7*^*R26Luc/+*^ mice challenged with 30 min IRI were given a s.q. dose of MitoT (bottom panel) or saline vehicle control (top panel). **b** H_2_O_2_ production was determined by quantifying kidney ROIs over kidney region and readout of PC-Luciferin activity compared with pre-IRI condition. Levels of H_2_O_2_ in MitoT treated animals at 24 h after IRI were not significantly different compared with time pre-IRI. *n* = 10 and 8 mice for IRI and IRI with MitoT cohorts. ***P < 0.001 between IRI and IRI treated with MitoT. ^##^*P* < 0.01 and ^####^*P* < 0.0001 vs respective time 0. **c** Pretreatment with MitoT significantly reduced MDA levels 27% (*P* = 0.0027 6 h after IRI). *n* *=* 4 and 8 kidneys for untreated sham and IRI, respectively, and *n* = 4 and 10 kidneys for treated sham and IRI, respectively. **d**, **e** Blood parameters, serum creatinine and BUN, were not significantly different at 24 h post IRI. **P < 0.01 between untreated and MitoT cohorts. ^###^*P* < 0.001 and ^####^*P* < 0.0001 vs. respective sham

Liberated iron complexes from mitochondria, necrotized cells and erythrocytes constitute ROS which subsequently oxidize cellular lipid and lipid released from necrotic tissue. Furthermore, mitochondria are a dominant source of cellular ROS^31^ and thus likely a critical driver of IRI in the collecting duct where mitochondria are abundant. To test this hypothesis, MitoTEMPO (MitoT), a mitochondria-specific superoxide scavenger, was used to investigate the role of ROS and in particular, mitochondrial ROS in IRI. A single bolus of 10 mg/kg of MitoT administered s.q. immediately prior to IRI surgery had a significant effect on H_2_O_2_ activity at 6 h (mean 57.5-fold vs 6.19-fold change; *P* *<* 0.001), and delaying the upregulation of ROS in the collecting duct till 12 h post IRI (mean 55.7-fold vs 34.9-fold with treatment: Fig. 5a, b). We did not observe a significant difference in sCr or BUN at 6 h (Fig. 5c, d) because blood metabolites do not change rapidly. However, MitoT was observed to protect the kidney from the other manifestations of IRI damage including inflammation and ROS lipid peroxidation as shown by the significant reduction in MDA levels 6 h (*P* = 0.0027) after IRI compared with the untreated group (Fig. 5c and Supplementary Table 3).

### Collecting duct is a significant site of oxidative damage

**Fig. 6:**
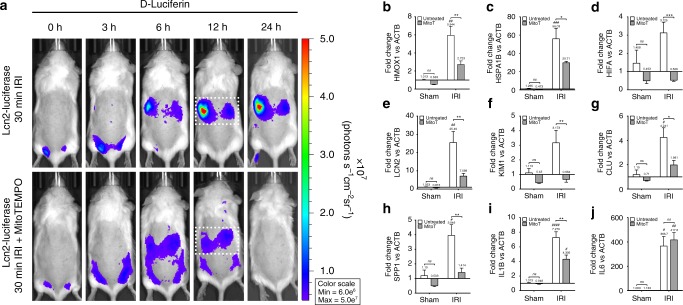
MitoTEMPO pretreatment reduces kidney injury, oxidative stress, and inflammatory biomarkers. Lcn2-luciferase reporter mouse. The expression of distal and collecting duct kidney injury biomarker, Lcn2-Luc, was reduced with single bolus of MitoT after 30 min IRI injury. Kidney region of interest in dotted bounding box. **a** Pretreatment with MitoT consistently reduced *Lcn2* expression (*P* *=* 0.0002) in 30 min IRI measured 24 h after insult.** b**–**d** At 24 h, Hmox1, Hspa1b, and Hif1a gene expression were significantly reduced by MitoT, indicating a reduction in oxidative stress. **e**–**h** Both cortical and medullary compartments were protected by MitoT treatment shown by significant reduction in kidney injury gene expression *Lcn2, Kim1*, *Clu*, and Spp1, respectively. **i** Inflammatory cytokine IL1b mRNA levels were significantly reduced in MitoT group compared with untreated IRI in contrast to **j** no reduction in *Il6* expression. (qPCR: *n* *=* 4 and 8 kidneys for untreated sham and IRI, respectively, and *n* *=* 4 and 10 kidneys for treated sham and IRI, respectively)

LCN2 is an established marker of injury expressed in the thick ascending loop of Henle and the collecting duct^15^. The *Lcn2*-reporter mouse has been shown to be a significantly more sensitive system to test the time course of kidney injury than sCr or BUN^15^. Furthermore, we have shown that LCN2 is an iron scavenger in septic conditions^32^ and likely in aseptic conditions as well^33^. Consequently, we challenged the *Lcn2*-luciferase reporter mouse^15^ with 30 min of bilateral IRI with and without MitoT and longitudinally imaged oxidative stress with PC-luciferin. With a single application of MitoT immediately prior to ischemic injury, *Lcn2*-luciferase expression in the MitoT group returned to baseline by 24 h post IRI, whereas the untreated group maintained 20-fold *Lcn2*-luciferase reporter activity over baseline (Fig. 6a). These data reveal that even a modest reduction of mitochondrial oxidative stress can suppress responses characteristic of renal cell injury.

In this model, we also found that highly sensitive and rapidly responding kidney injury-associated genes showed significant (*P* < 0.05) reduction following MitoT treatment (Fig. 6b–j). Oxidative stress transcripts Hmox1, Hspa1b, and Hif1a were significantly (*P* < 0.05) downregulated (Fig. 6b–d) as was a panel of established kidney injury response genes (Fig. 6e–g). Furthermore, the inflammatory potential of IRI was reduced as shown by the significant (*P* < 0.05) reduction in pro-inflammatory cytokine expression (Fig. 6i and Supplementary Table 5)^34^. Interestingly, Il1b improved while Il6 was not ameliorated, suggesting reduced activation of the inflammasome (Fig. 6j)^35^.

## Discussion

Many acute human tissue injuries are identified through changes in the function of the target tissue, for example IRI results in decreased filtration which results in a rise in serum Creatinine (sCr) and blood urea (BUN). However, these clinically identifiable markers of tissue injury may be detected in only a fraction of injuries, are not well correlated with injury severity, and can have slow kinetics^21,36^. Indeed, sCr does not reflect renal pathology in the intensive care unit^37,38^, nor does sCr necessarily correlate with urinary biomarkers since the latter are expressed earlier than the rise in sCr and with greater sensitivity and specificity for tissue damage. In animal models, changes in blood chemistry, histology, and gene expression can all be used to identify and study injury response. However, these tools have limited temporal resolution, are invasive, and generally terminate the experiment. There is need for an approach which allows monitoring of the injury response non-invasively in laboratory animals in vivo.

In order to understand the anatomy and physiology of tissue injury, we instituted two different types of imaging technology. The first provides tissue specificity. The heterogeneous composition of the kidney is not typically considered when monitoring AKI by standard clinical tests or even when urinary biomarkers are measured by quantitative tests such as Elisa. It is also difficult to distinguish the contribution of each cell-type when interrogating the organ as a whole or to know which molecular pathway is primary or secondary to damage. A number of cell-specific animal models have become available to researchers^39^ and when combined with luciferase-based technology^15^, longitudinal and repetitive assessment of kidney injury is possible. Here we instituted a variety of tissue-specific Cre-recombinase mouse lines to provide a genetic means of activating luciferase in different compartments of the kidney.

The second tool that is necessary for physiologic interrogation is an assortment of biologically relevant reporters that monitor different cellular events. Caged-luciferin chemistry enables the interrogation of numerous processes with enzymatic activities such as cell-penetrating peptide conjugates^40^, cell surface glycosylation^41^, hydrogen peroxide fluxes^1^, and fatty acid uptake^42^. It also allows imaging of enzyme activities such as beta-galactosidase^43^, caspases^44–46^, furin^47^, and beta-lactamase^48^ in the presence of firefly luciferase. Hence this broad range of substrates can effectively map the kinetics of many different cellular functions in various forms of tissue damage or after treatment with protective medications. The broad applicability of this technique is demonstrated by caspase 3/7 caged-luciferin expression after IRI (Supplementary Fig. 8)^49^.

By combining the caged-luciferin technology with tissue-specific Cre-mediated luciferase, we obtained tissue-specific readouts of the activated form of PC-Luciferin to monitor the generation of a key ROS molecule, H_2_O_2_ after IRI. Surprisingly the collecting duct was a major source of H_2_O_2_ based on PC-Luciferin activated in the HoxB7-luciferase mouse. One-third of the cells of the collecting duct are mitochondrial rich intercalated (IC) cells, suggesting that these cells may be the primary source of the H_2_O_2_. We confirmed the mitochondrial basis of collecting duct responses to IRI by quantitative Bio-Imaging which identified multi-phasic production of ROS, highlighting informative time points for cell-specific *HoxB7*-RiboTRAP RNA-seq analysis. In turn, this approach guided our transcriptomics time point selection and RNA-seq analysis revealed key transcriptional responses that regulated the function of mitochondria and generated ROS, in particular, we identified downregulation of mitochondrial complex I (Cx1). Mitochondrial dysregulation is likely to be a critical mechanism for collecting duct injury because the IRI phenotype was ameliorated with a single bolus of a mitochondrial antioxidant (MitoTEMPO), which significantly reduced acute lipid peroxidation and decreased the expression of the kidney injury biomarkers Ngal and Kim1 (which may secondarily reflect collecting duct dysfunction). In sum, H_2_O_2_ generation, lipid peroxidation, biomarkers of injury and cytokine expression all correlated with, and were dependent on ROS generation in the collecting duct.

Liberated iron complexes from mitochondria, necrotized cells and erythrocytes make ROS which subsequently oxidize cellular lipid and lipid released from necrotic tissue. Furthermore, mitochondria are a dominant source of cellular ROS^31^ and thus likely a critical driver of IRI, in the collecting duct where mitochondria are abundant. MitoTEMPO as an antioxidant of the extracellular space may also play a role in the reduction of the observed lipid peroxidation of the kidneys. In this case, it is likely that mitoTEMPO is suppressing extracellular ROS activity due to liberated iron complexes from necrotized cells and erythrocytes. These ROS species are suppressed by the antioxidant activity of MitoTEMPO in the extracellular space and the reduction of the collecting duct kidney injury reporter and iron-scavenger LCN2 is reduced. In addition, the reduction of Kim1 expression, a proximal tubule injury reporter, indicates that kidney injury as a whole was reduced in the MitoTEMPO condition. These data support our previous findings that LCN2 can operates as an iron scavenger in aseptic conditions and is a sensitive reporter of kidney injury^15,33^.

Differences between collecting duct and proximal tubule response to IRI was apparent in our studies. The collecting duct ROS reporter and biomarker expression were dose responsive; 30 min of ischemia resulted in a larger signal than 15 min. Expression of the collecting duct injury marker Lcn2 and the distal nephron injury marker Tacstd2 were also dose responsive. In contrast, the proximal tubule showed maximal ROS signal responsiveness after 15 min of ischemia; doubling the ischemia time did not further increase the response. This result is consistent with the expression of the proximal tubule-specific injury biomarker Kim1 which is higher following 15 min of IRI than 30 min (Supplementary Fig. 9). ROS are generated by multiple mechanisms in vivo and have multiple functions and toxicities. ROS can be generated both by released heme, for example from necrotically damaged cells and mitochondria, and by a genetic program that alters mitochondrial function as well as altered expression of the extracellular peroxide pumps, the NOX complexes. ROS also act as signals of injury and an antipathogen function. Our transcriptomics data are consistent with the hypothesis that the collecting duct is actively generating ROS as part of its injury response. For example, the changes in expression of Cx1, and NOX suggest that collecting duct cells are actively generating increased ROS, perhaps as a component of their barrier function to inhibit bacterial growth or as a component of an injury signaling system. Consistent with this observation, we have shown collecting duct cells in response to a urinary tract infection also decrease the urinary pH and increase expression of the iron scavenger LCN2 (Paragas et al., *JCI)*^32^.

Alternatively, the threshold effect for activation of the collecting duct may be due to a robust antioxidant scavenging system that requires 30 min of ischemia to achieve saturation before demonstrating lipid peroxidation and log order increases in tissue-specific H_2_O_2_ generation. Conversely, it may be that apoptosis, necrosis and/or cellular dysfunction of proximal tubule cells^50^ results in saturation of its dynamic range to generate H_2_O_2_ and Kim1 at higher levels of IRI whereas the collecting duct, in contrast, is not subject to cell death and instead responds in a dose-responsive manner to a greater range of IRI injury^15^. This model solves a long-standing debate as to the timing of which segment is affected in AKI and is consistent with previous findings concerning the dose response of collecting duct vs proximal tubule biomarkers in both mice and humans and to the varied histologic findings of different tubular segments in the AKI kidney^51–54^.

This study presents a new and powerful integrated framework to dynamically associate pathophysiological changes to transcriptional signals at the cellular level. However, it is only the glimpse into the kidney’s reaction to an acute injury, because the kidney has a great cellular heterogeneity and there are many different models of kidney injury to map. We used *HB7*^*R26Luc*/+^, *Slc34a1*^*R26Luc/+*^, and *Pod*^*R26Luc/+*^ genetic models to map ROS activity in a subset of cells representing the cortex and medulla of the kidney. Clearly, there is a need to expand on this work and investigate the effect of different types of renal injury on the transcriptional responses of other segments of the nephron and distal tubule in order to discover common biomarkers and therapeutic targets in AKI.

In this paper we present an ‘image-guided omics’^55^ technology platform to monitor AKI progression through non-destructive tools. Our approach provides a system to temporally study kidney injury, gives insight into how the kidney utilizes endogenous mechanisms to regulate injury pathways, and allows testing and discovery of novel therapeutic interventions targeting ROS signaling pathways. Our system is a cell-specific, longitudinal, non-destructive, and non-invasive method to study of ROS dynamics using PC-luciferin in a novel application^1^. In contrast, other imaging systems such as multiphoton microscopy^56^ permit imaging only at a depth of a few millimeters from the lens, which limits visualization of the medulla. Further, they require animal surgery during the data acquisition phase, which may contribute to marked variability in the findings and they do not allow longitudinal monitoring over hours because of repetitive stress on the animal. Our non-invasive system is a log order more sensitive compared with traditional methods of ROS measurements by MDA.

LCN2 from the thick ascending limb and the collecting duct presaged the idea that these compartments respond to IRI^15^, here we visualized the collecting duct response in real-time and demonstrated that underlying mitochondrial genes are important components of this pathologic readout. In addition, we found that damage biomarkers correlated with the time course of ROS rather than the time course of serum creatinine, again highlighting the distinction between gross functional changes and segment specific molecular mechanisms. However, our findings have wider clinical implications beyond the kidney since application of these novel tools can help identify and test therapeutics targeting oxidative stress in many cell types spanning different organs. In each of these applications, the proposed luciferase system can be scaled to assess multiple physiological readouts, enabling the identification of meaningful disease endotypes within complex disorders (such as AKI) based on spatiotemporal functional mapping of distinct pathophysiologic and molecular events.

## Methods

### Mice

*ROSA26 L-S-L-Luc/+*[*FVB*.*129S6(B6)-Gt(ROSA)26Sortm1(Luc)Kael/J*, 005125] mice were bred with *Aqp2-Cre* [*B6*.*Cg-Tg(Aqp2-cre)1Dek/J*, 006881], EIIa-Cre [*B6*.*FVB-Tg(EIIa-cre)C5379Lmgd/J*, 003724], *HoxB7-Cre* [Tg(Hoxb7-cre)13Amc/J, 004692], Podocin-cre [B6.Cg-Tg(NPHS2-cre)295Lbh/J, 008205] obtained from Jackson Labs, *Lcn2*-Luciferase reporter from J.M.B., *Slc34a1-CreER(T2)* from A.K., and *Gt(ROSA)26Sor-mCherry-Rpl10a* from EMMA. *Slc34a1-CreER(T2)* expression is activated by tamoxifen administration. Briefly, tamoxifen was dissolved in filtered corn oil (20 mg/mL) at 37 °C and injected i.p. (100 mg/kg) every other day for 4 days using 8-week mice. After breeding the conditional luciferase expressers are known as *EIIa*^*R26Luc/+*^, *Pod*^*R26Luc/+*^, *Slc34a1*^*R26Luc/+*^, *HoxB7*^*R26Luc/+*^, and *Cdh5*^*R26Luc/+*^. C57BL/6 (13 week) were obtained from Jackson Labs. Mice were single or group-housed on a 12∶12 light–dark cycle at 22 °C with food and water ad libatum. Ten- to sixteen-week-old male mice were used for all IRI experiments as previously published^15^. All animal studies were approved by and performed according to the guidelines approved by the Institutional Animal Care and Use Committee (IACUC) at University of Washington.

### Cell-specific RiboTRAP animal

To determine gene expression in a single cell-type at a single time point during a AKI we bred the *Gt(ROSA)26Sor-mCherry-Rpl10a* (mCherryTRAP) mouse that labels ribosomes for immunoprecipitation isolation of RNA with a tissue-specific Cre-recombinase mouse^57^. *HoxB7-Cre* (*HoxB7*^*TRAP/+*^) was used to label ribosomes in the collecting duct. This strategy relies on Cre-recombinase dependent activation of a mCherry-Rpl10a ribosomal protein subunit, which allows translating ribosome affinity purification (TRAP) of mRNA populations in Cre-expressing cells. Immunostaining of RPL-mCherry and immune-precipitated RNAs demonstrated specific enrichment of HoxB7^TRAP/+^ (CD) populations by confocal microscopy and qPCR (Fig. S7).

### Purification of mRNA from tissue mCherry/TRAP mice

Kidneys from RiboTRAP mice were harvested, flash-frozen in liquid nitrogen and stored in −80 °C until homogenization. Frozen kidneys were homogenized and the lysate was added to Anti-RFP magnetic beads (MBL International, WA) and mCherry/TRAP bound beads were separated from the supernatant with a magnet (Fig. 4). RNA was eluted and purified using RNeasy Micro Kit (Qiagen) according to the manufacturer’s instructions. RNA integrity number (RIN) was measured using the Agilent 2200 TapeStation (Agilent Technologies, TX) and only samples with a RIN than or equal to 6.8 was used. qPCR was used to validate relative enhancement of tissue-specific genes of the collecting duct (Aqp2).

### RNA-sequencing

HiSeqLibrary was generated with 950 ng of mRNA using Illumina’s TruSeq RNA with Ribo-Zero protocol (Illumina, CA). RNA-seq (Illumina HiSeq) was performed at UW Cystic Fibrosis Genomics Core Center, followed by image analysis and base calling with Illumina’s RTA (v1.18) software. Demultiplexing and FASTQ format file generation and alignment was performed using HiSeq analysis software (TopHat v2.1.1) with UCSC mouse genome build mm10. Quality control was then performed with RNA-SeQC v1.1.8 and counts generated using HTSeq-counts v0.7.2. Differential gene expression between IRI and sham conditions was determined using DESeq2 package in R environment^58^ using a false discovery rate (FDR) cutoff <0.01. Functional enrichment of differentially expressed genes was performed using Webgestalt program^59^ and was based on pathways and processes from multiple resources, including KEGG, Reactome, and Gene Ontology (GO). An enrichment FDR < 0.01 was used to designate significant overrepresentation of functional categories and pathways.

### Kidney ischemia-reperfusion injury (IRI) model

The renal artery was clamped using vascular clamps for a period of 30 min according to an established model of warm IRI to induce ischemic acute renal failure^15^. Briefly, 10–16-week-old male mice were anesthetized with gas anesthesia (isoflurane). The body temperature was maintained at 37 °C throughout the procedure. Kidneys renal pedicles were clamped for 30 min using nontraumatic microaneurysm clips (Fine Science Tools). Restoration of blood flow was monitored before closing incisions with surgical staples. Sham-operated mice underwent the same procedure except for clamping of the arteries. MitoTEMPO (Sigma) was suspended in sterile saline to 2.5 mg/ml and given s.q. immediately prior to IRI surgery.

*In vivo animal imaging*: An IVIS Spectrum instrument (PerkinElmer) was used for bioluminescent imaging in all animal experiments. Mice were anesthetized prior to injection and during imaging via inhalation of isoflurane (Phoenix Pharmaceuticals, Inc.) mixed with medical-grade oxygen (Praxair). Validation of luciferase expression was done using the potassium salt of D-luciferin (PerkinElmer) resuspended in PBS from ThermoFisher Scientific and injected i.p. (150 mg Luciferin/kg body weight).

### Image data analysis

Planar bioluminescent images were quantified using LivingImage (PerkinElmer) software and tomographic bioluminescent images with InVivoAX (InVivo Analytics, Inc.) software. For kinetic image analysis of caged-luciferin with LivingImage, bioluminescent kidney radiance (photons s^−1^ cm^−2^ sr^−1^) was measured by manually drawing two equally sized circular ROIs (1 cm in diameter) over both the left and right kidney. Each kidney was treated as a separate data point. PC-Luciferin signal acquired in an untreated animal was used throughout the experiment as the baseline for delta change in radiance and as the denominator for fold-change calculations. The delta in total photon flux for each condition was calculated by dividing the total photon flux for the experimental condition by the total photon flux for the pre-IRI alone to allow a direct comparison between various kidney-specific luciferase reporter animals. Kinetic curves of *HoxB7*-luciferase, *Slc34a1*-luciferase and *Pod*-luciferase calculated with images acquired every 3 min (*n* = 20) starting 1 min post D-luciferin injection.

### Bioluminescent tomographic reconstruction

Animals placed body conforming animal mold (InVivo Analytics, Inc.) and multi-view multi-spectral image acquired with the InVivoPLOT mirror-gantry. Multi-view multi-spectral images were reconstructed using InVivoAX (InVivo Analytics, Inc.) cloud-based software and aligned to digital mouse atlas. Photon image density was determined using InVivoAX (InVivo Analytics, Inc.) software. The source density is the amount of emitted photons per unit volume and time unit (photons cm^−3^ s^−1^). The relative source density method permits accurate and quantitative comparisons across all image data sets.

### In vivo ROS monitoring

Mice were i.p. injected with PC-luciferin (0.5 μmol in PBS) as previously described in Van Bittner et al.^1^. Following injections, mice were imaged with an IVIS Spectrum for 1 h to capture the peak bioluminescent signal generated by uncaged PC-luciferin. PC-luciferin probe free acid was prepared according to published procedure^1^ starting from 6-hydroxybenzo[*d*]thiazole-2-carbonitrile (ABCR), 2-(4-(bromomethyl)phenyl)-4,4,5,5-tetramethyl-1,3,2-dioxaborolane (TCI), D-cysteine (ABCR). The product was purified by preparative HPLC (RP silica with a gradient over 10 min of 5–100% acetonitrile in water), converted into potassium salt by a reaction with potassium bicarbonate (Sigma Aldrich) in methanol–water 2:1 mixture, and finally lyophilized to give PC-luciferin as a white powder. The change in total photon flux for each condition was calculated by dividing the total photon flux for the experimental condition ROI by the total photon flux for PCL-1 pre-IRI condition ROI to allow a direct comparison between various kidney-specific luciferase reporter animals. Clearance of PCL-1 probe was determined by imaging E2a-Luciferase mice over 3 h with PC-luciferase (0.5 μmol in PBS). ROS activity and PC-Luciferin clearance monitored by response to hydrogen peroxide challenge (24 mM in 100 µl of PBS) before imaging and 2 h after first injection. Hydrogen peroxide challenge was consistent with work described in Van Bittner et al.^1^.

### Real-time PCR analysis

Total RNA was isolated with Trizol (Invitrogen), quantified by NanoDrop Spectrophotometer and the first strand cDNA was synthesized using High-Capacity cDNA Reverse Transcription Kit (Applied Biosystems). Real-time PCR was performed to quantify Ngal/Lcn2 (Mm.PT.58.10167155), Kim1/Havcr1 (Mm.PT.58.31303329), Hmox1 (Mm.PT.58.8600055), IL1a (Mm.PT.58.32778767), IL18 (Mm.PT.58.42776691), Umod (Mm.PT.58.7038212), Clu (Mm.PT.58.11183004), Egf (Mm.PT.58.6246680), Gsta4 (Mm.PT.58.17030386), Hspa1b (Mm.PT.58.31570020.g), Spp1 (Mm.PT.58.43709208), Vegfa (Mm.PT.58.14200306), Hif1a (Mm.PT.58.11211292), Tnf (Mm.PT.58.12575861), Mx1 (Mm.PT.58.12101853.g), IL1b (Mm.PT.58.41616450), IL6 (Mm.PT.58.10005566), C3 (Mm.PT.58.17325540), C1q (Mm.PT.58.7482171), Ifit1 (Mm.PT.58.32674307), and bACT (Mm.PT.39a.22214843.g) mRNA expression in a LightCycler 96 (Roche) qPCR system with PrimeTime® Gene Expression Master Mix (IDT) and probe-based PrimeTime® qPCR Assays (IDT). β-actin was quantified as the housekeeping gene. The 2^ΔΔCT^ method was used to calculated fold amplification of transcripts.

### Measurement of lipid peroxides

The levels of lipid peroxides (LPOs) in each kidney were determined by measuring malondialdehyde (MDA) content using the TBARS (TCA Method) Assay Kit (Cayman Chemical Company) according to manufacturer’s instruction. Briefly, fresh kidney was bisected (other half used for qPCR) and homogenized in RIPA buffer (Alfa Aesar) containing protease inhibitor (cOmplete, Mini, Roche) at a ratio of 25 mg of tissue in 250 µl of RIPA buffer. Half of left and right kidney was processed for each mouse. The tissue was homogenized on ice using glass dounce, centrifuged at 1600 × *g* for 10 min at 4 °C, and the supernatant was used for both the MDA and protein analyses. Absorbance was read at 535 nm and calculated using the derived standard curve. Protein levels were measured using a Qubit Protein Assay Kit (ThermoFisher Scientific) according to manufacturer’s instruction. MDA was normalized in each sample in relation to total protein concentration and data are presented at nmol MDA/mg protein.

### Blood parameters

Heparinized blood was analyzed using the clinical blood analyzer iStat (Abbott). The Crea and the EC8 + cartridges were used for creatinine and Na, K, Glu, pH, PCO_2_, BUN/Urea, Hct, HCO_3_, TCO_2_, BE, AnGap, and Hb, respectively.

### Statistics and reproducibility

Data were presented as mean ± SEM. GraphPad Prism software, version 6.0c (GraphPad Software Inc., San Diego, CA) was used for statistical analyses. Repeated measures one-way ANOVA with Dunnett’s multiple comparisons test was used to compare the luminescence data. One-way ANOVA with Dunnett’s multiple comparisons test or two-way ANOVA with Tukey’s multiple comparisons test was used to compare BUN, creatinine and MDA levels. One-way ANOVA with Dunnett’s multiple comparisons test or two-way ANOVA with Tukey’s multiple comparisons test was used to compare qPCR. *P*-value < 0.05 was considered a statistically significant difference.

### Reporting summary

Further information on research design is available in the Nature Research Reporting Summary linked to this article.

## Supplementary information

Supplementary Information

Description of additional supplementary items

Supplementary Data 1

Supplementary Movie 1

Supplementary Movie 2

Supplementary Movie 3

Reporting Summary

## Data Availability

Sequence data that support the findings of this study have been deposited in the Gene Expression Omnibus (GEO) with the primary accession code GSE133184. All other data can be obtained from the corresponding author upon request.
