# Supplementary material for: Cell-specific image-guided transcriptomics identifies complex injuries caused by ischemic acute kidney injury in mice
Source: Commun Biol. 2019 Sep 2;2:326. doi: 10.1038/s42003-019-0571-7 (PMC6718519; doi:10.1038/s42003-019-0571-7)
Supplement: Supplementary file 1 — Supplementary Information [file 42003_2019_571_MOESM1_ESM.pdf]

## Supplementary Figure 1

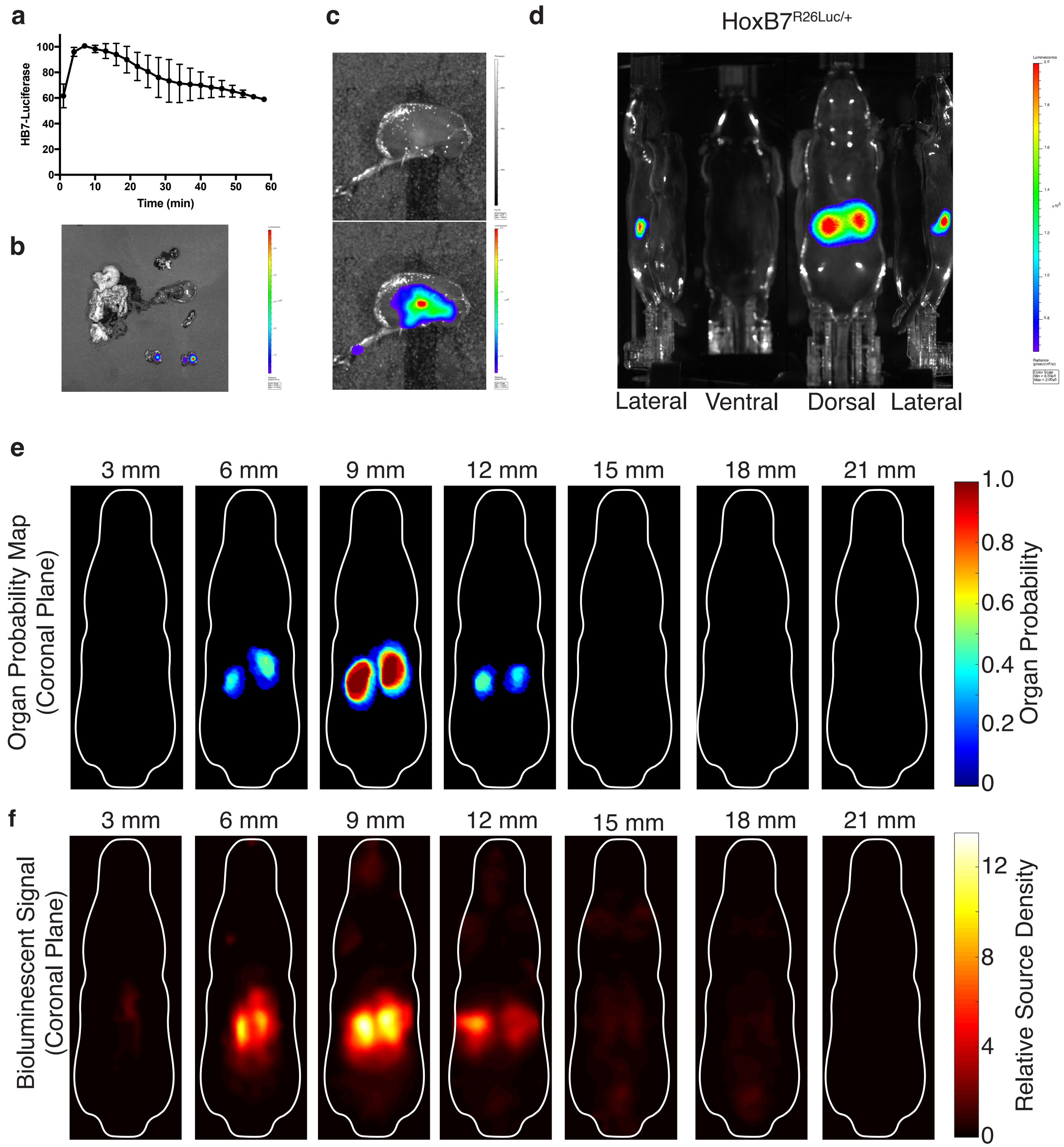


**Supplementary Figure 1. Bioluminescent tomographic reconstruction confirms kidney location of collecting duct (*HoxB7*) luciferase activity.** Nephron-specific luciferase reporter activity was acquired in an optical imaging system. The kidney-specific luminescence derived from D-luciferin (150mg/kg) was validated by bioluminescent tomography aligned to a digital mouse atlas. **a** Whole animal bioluminescent kinetics with D-luciferin acquired every 3 minutes over 60 min. **b** Excised internal organs after D-luciferin injection. **c** Bisected kidney close-up with photograph (top panel) and overlay with bioluminescent image (bottom panel). **d** *HoxB7*$^{R26Luc/+}$ image acquired with a four-view mirror gantry, InVivoPLOT (InVivo Analytics, Inc.). Mirror-gantry simultaneously acquires dorsal, ventral and both lateral views. Kidney luminescent signal visible in dorsal and both lateral views. **e** Bioluminescent tomographic reconstruction performed (InVivoPLOT; InVivo Analytics Inc.), **f** aligned with digital mouse atlas (Organ Probability Map; InVivo Analytics Inc.) for the kidney.

## Supplementary Figure 2

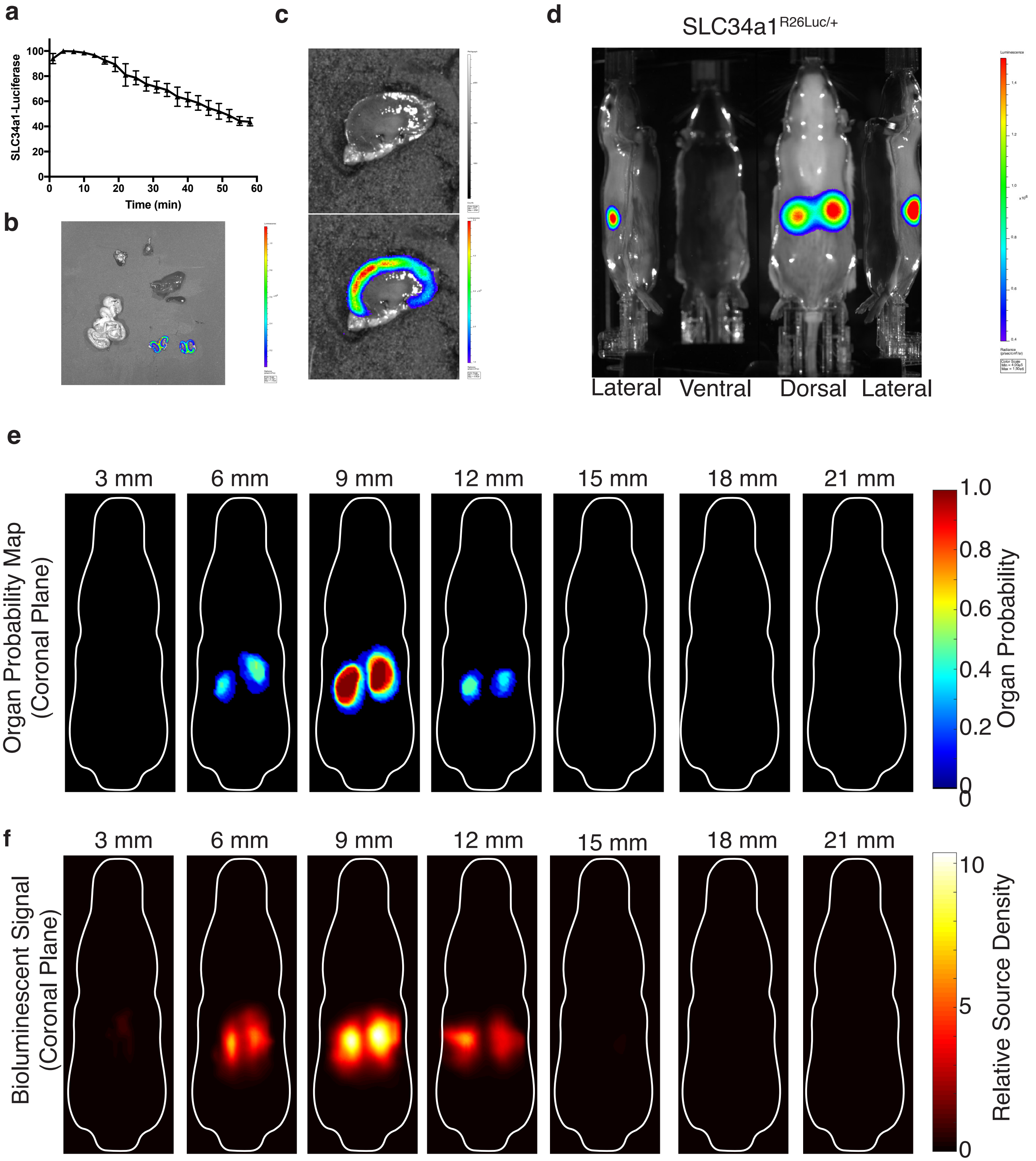


**Supplementary Figure 2. Bioluminescent tomographic reconstruction confirms kidney location of proximal tubule (*Slc34a1*) luciferase activity.** Nephron-specific luciferase reporter activity was acquired in an optical imaging system. The kidney-specific luminescence derived from D-luciferin (150mg/kg) was validated by bioluminescent tomography aligned to a digital mouse atlas. **a** Whole animal bioluminescent kinetics with D-luciferin acquired every 3 minutes over 60 min. **b** Excised internal organs after D-luciferin injection. **c** Bisected kidney close-up with photograph (top panel) and overlay with bioluminescent image (bottom panel). **d** *Slc34a1*$^{R26Luc/+}$ image acquired with a four-view mirror gantry, InVivoPLOT (InVivo Analytics, Inc.). Mirror-gantry simultaneously acquires dorsal, ventral and both lateral views. Kidney luminescent signal visible in dorsal and both lateral views. **e** Bioluminescent tomographic reconstruction performed (InVivoPLOT; InVivo Analytics Inc.), **f** aligned with digital mouse atlas (Organ Probability Map; InVivo Analytics Inc.) for the kidney.

## Supplementary Figure 3

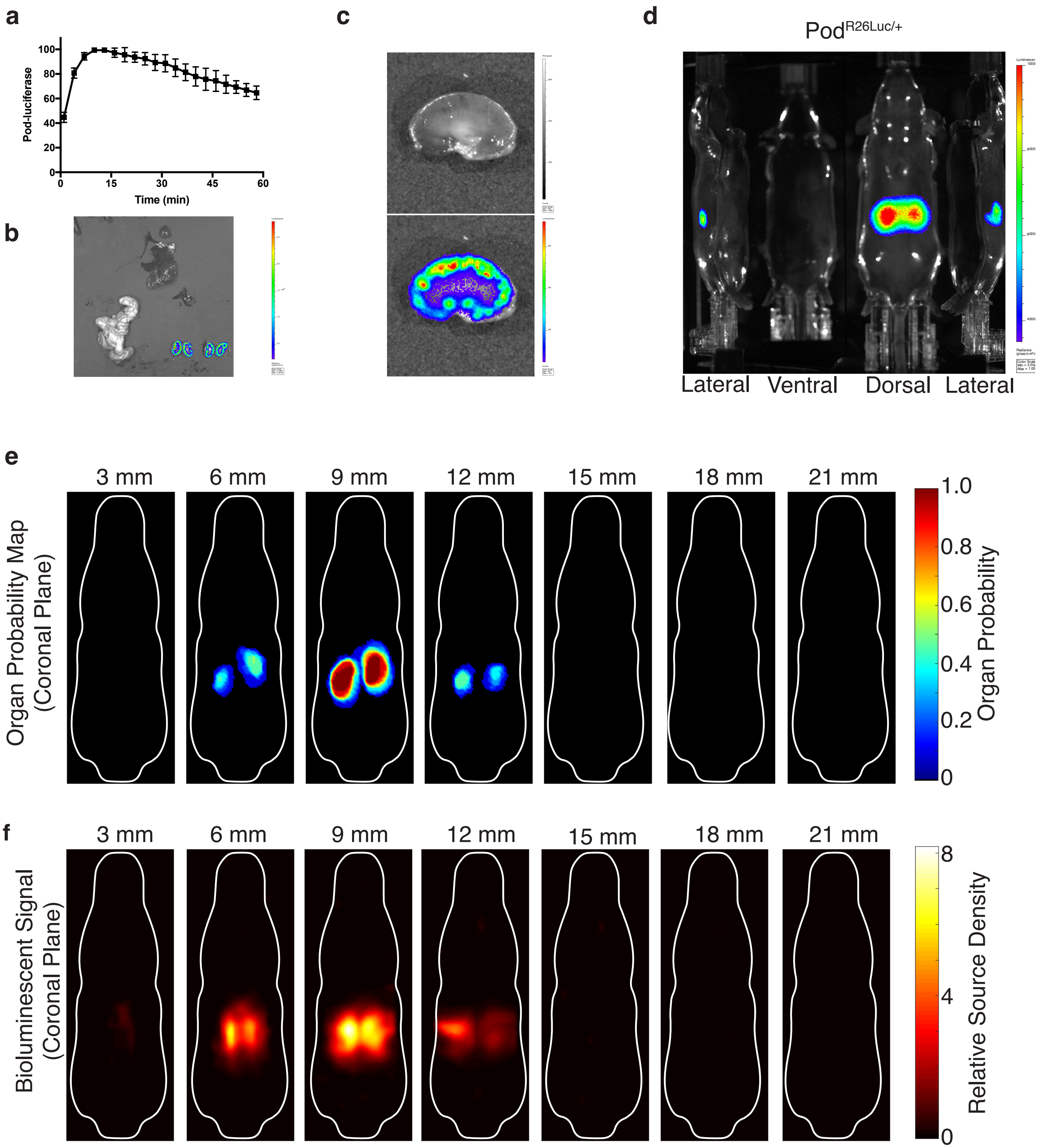


**Supplementary Figure 3. Bioluminescent tomographic reconstruction confirms kidney location of glomerular (*Pod*), luciferase activity.** Nephron-specific luciferase reporter activity was acquired in an optical imaging system. The kidney-specific luminescence derived from D-luciferin (150mg/kg) was validated by bioluminescent tomography aligned to a digital mouse atlas. **a** Whole animal bioluminescent kinetics with D-luciferin acquired every 3 minutes over 60 min. **b** Excised internal organs after D-luciferin injection. **c** Bisected kidney close-up with photograph (top panel) and overlay with bioluminescent image (bottom panel). **d** *Pod*$^{R26Luc/+}$ image acquired with a four-view mirror gantry, InVivoPLOT (InVivo Analytics, Inc.). Mirror-gantry simultaneously acquires dorsal, ventral and both lateral views. Kidney luminescent signal visible in dorsal and both lateral views. **e** Bioluminescent tomographic reconstruction performed (InVivoPLOT; InVivo Analytics Inc.), **f** aligned with digital mouse atlas (Organ Probability Map; InVivo Analytics Inc.) for the kidney.

## Supplementary Figure 4

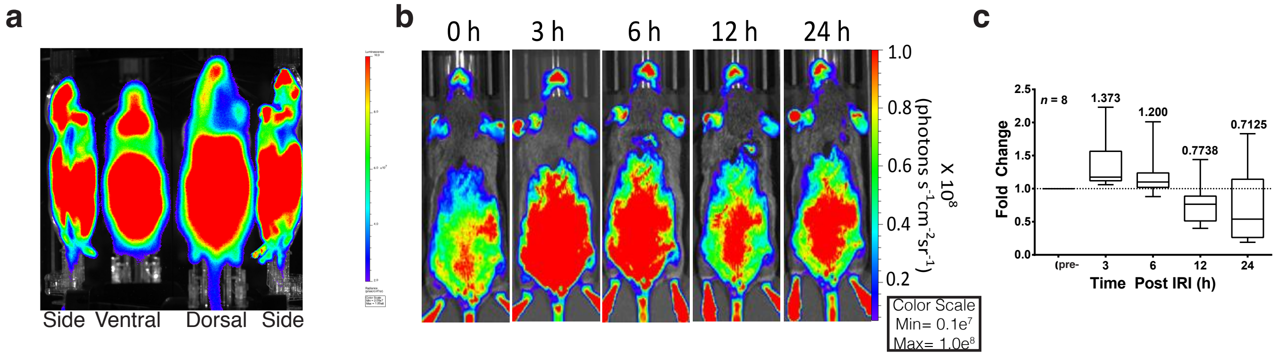


**Supplementary Figure 4. Global luciferase activity time course not effected by IRI. a** Baseline luciferase activity for EIIa-luciferase reporter (global) acquired in multiview mirror gantry using D-luciferin substrate. **b** Planar bioluminescent image acquired at pre-AKI, 3, 6, 12 and 24 h after AKI. **c** No significant kidney luminescent fold change observed from kidney ROIs 3, 6, 12, and 24h after AKI compared to pre-AKI.

## Supplementary Figure 5

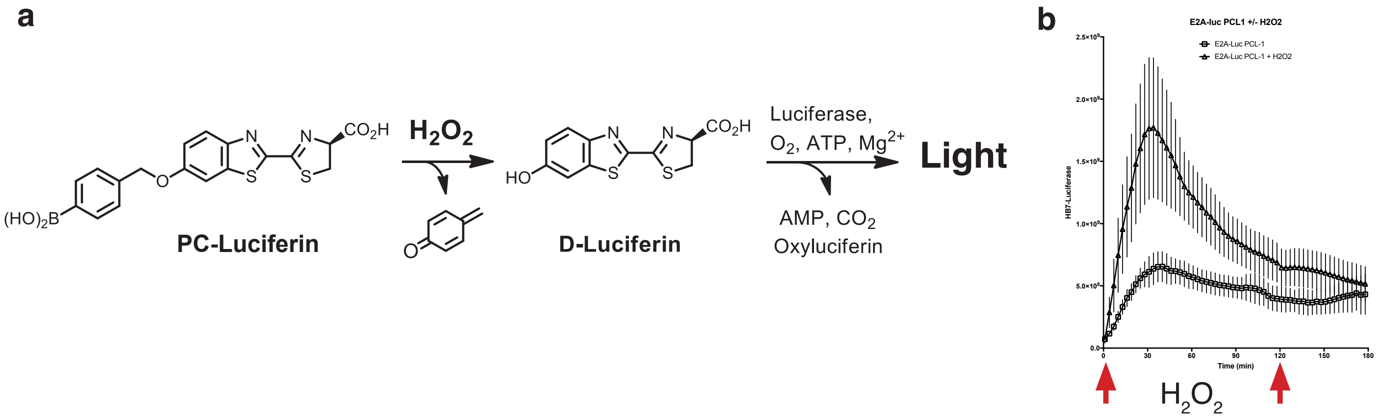


**Supplementary Figure 5. Chemistry and kinetics of Peroxy-Caged Luciferin (PC-Luciferin).** PC-Luciferin design strategy for selective H2O2-mediated light production. **a** PC-Luciferin is comprised of an $H_2O_2$-sensitive boronic acid attached to the phenolic oxygen of firefly luciferin molecule through a self-immolative benzylic linker. In the presence of endogenous $H_2O_2$, the boronic acid undergoes oxidative hydrolysis, yielding an unstable p-hydroxybenzyl luciferin that rapidly decomposes into D-luciferin. However, addition of $H_2O_2$ triggers selective cleavage of the "caged" moiety, leading to the release of free luciferin and production of a two-step oxidative reaction generating a bioluminescent signal proportional to the amount of $H_2O_2$. This PC-Luciferin was formulated as potassium salt to improve the solubility. **b** Additionally, we observed that once uncaged, PC-Luciferin is insensitive to $H_2O_2$. EIIa-Luciferase mice had ROS activity induced with a single i.p. bolus of $H_2O_2$ in PBS [24nM] and kinetic images acquired for 3 h with PC-luciferase (closed triangle). After 2 h, a second i.p. bolus of $H_2O_2$ was given to reveal remaining uncleaved PC-Luciferase. Endogenous ROS activity monitored by EIIA-Luciferase reporter animals without $H_2O_2$ (closed square) shows endogenous ROS activity. n = 3 for both conditions. ($H_2O_2$ i.p. 24 mM in 100 µL of PBS).

## Supplementary Figure 6

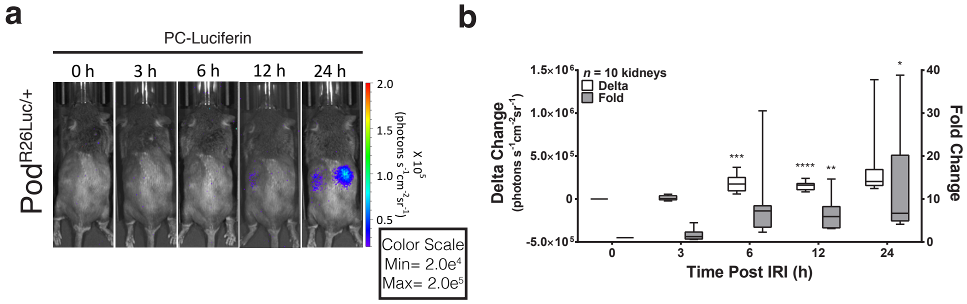


**Supplementary Figure 6. Glomerular specific non-invasive monitoring of $H_2O_2$ generation after acute renal injury. a** Glomerular compartment, reported by podocin-luciferase ($Pod^{R26Luc/+}$), **b** had significant fold changes at 12 and 24 h after 30 minutes of IRI and significant changes in absolute light emission at 6 and 12 h after injury. *n* = 10 mice for both.

## Supplementary Figure 7

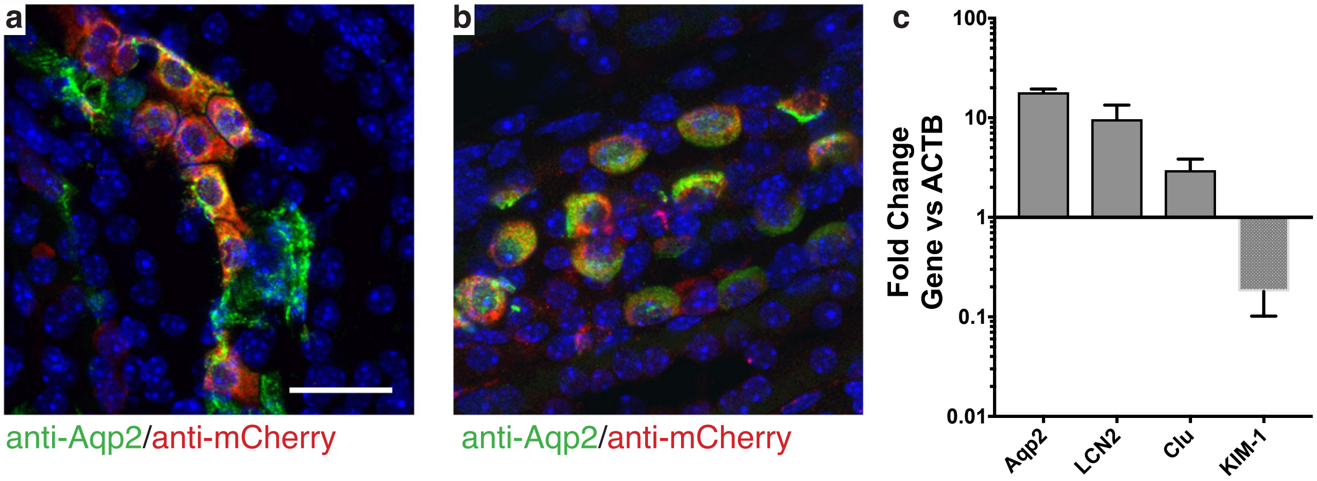


**Supplementary Figure 7. Validation of cell specific mCherry-RiboTRAP animals of the collecting duct. a** Kidney specific *HoxB7*-mCherry RiboTRAP kidney sections stained with anti-Aqp2 (green) and anti-mCherry (red). Co-staining confirms presence of mCherry-tagged ribosomes in the principal cell of the collecting duct. Magnification 40x. **b** Kidney specific *HoxB7*-mCherry-RPL kidney sections stained with anti-vATPase and anti-mCherry. Co-staining confirms the presence of mCherry-tagged ribosomes in intercalated cells of the collecting duct. Magnification 40x. **c** qPCR validation of *HoxB7*-mCherryRiboTRAP total kidney RNA versus magnetically immuno-precipitated RiboTRAP RNA. Collecting duct products *Aqp2*, *Lcn2* and *Clu* were enriched while KIM1 was diminished using the RiboTRAP method.

## Supplementary Figure 8

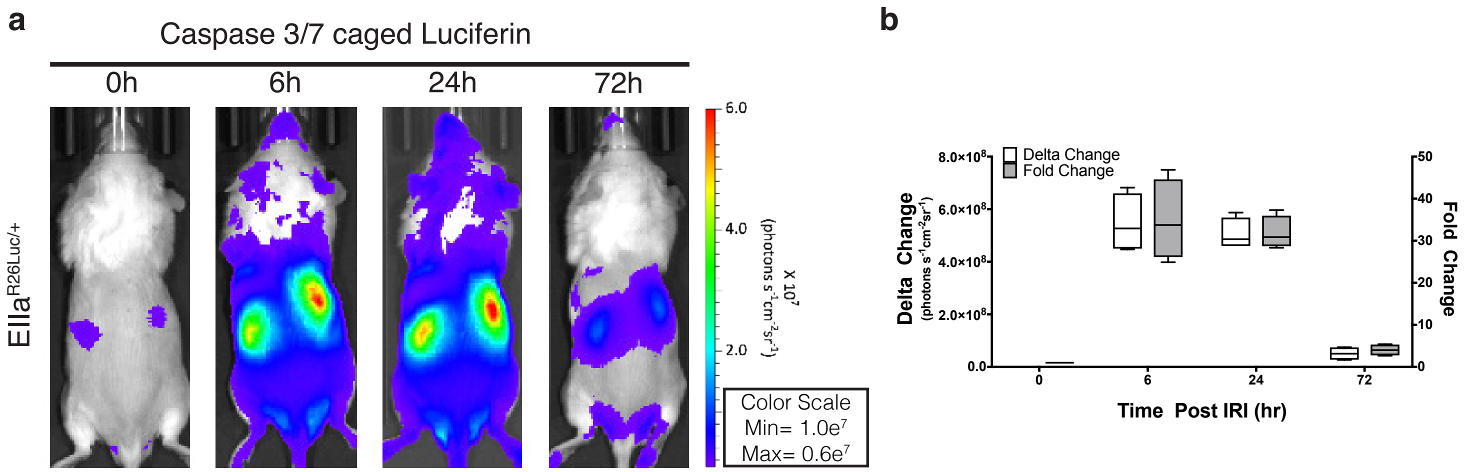


**Supplementary Figure 8. Caspase 3/7 spatial and temporal expression pattern after IRI. a** Global luciferase reporter model challenged with 15 min IRI and monitored with caspase 3/7 caged-luciferin revealed robust caspase 3/7 activity in the kidneys 6 h after IRI. **b** Fold and delta change of kidney caspase 3/7 activity. Caspase 3/7 activity was determined by quantifying kidney ROIs over kidney region and readout of caspase 3/7 activity compared to pre-IRI condition. n = 4 kidneys. Key genes of the apoptotic pathway in the collecting duct, Casp 3, 7, and 8 and Fas were found to be upregulated (1.35-fold ($P < 0.001$), 1.09-fold ($P = 0.5$), 1.64-fold ($P < 0.0001$)) 9 h after IRI.

## Supplementary Figure 9

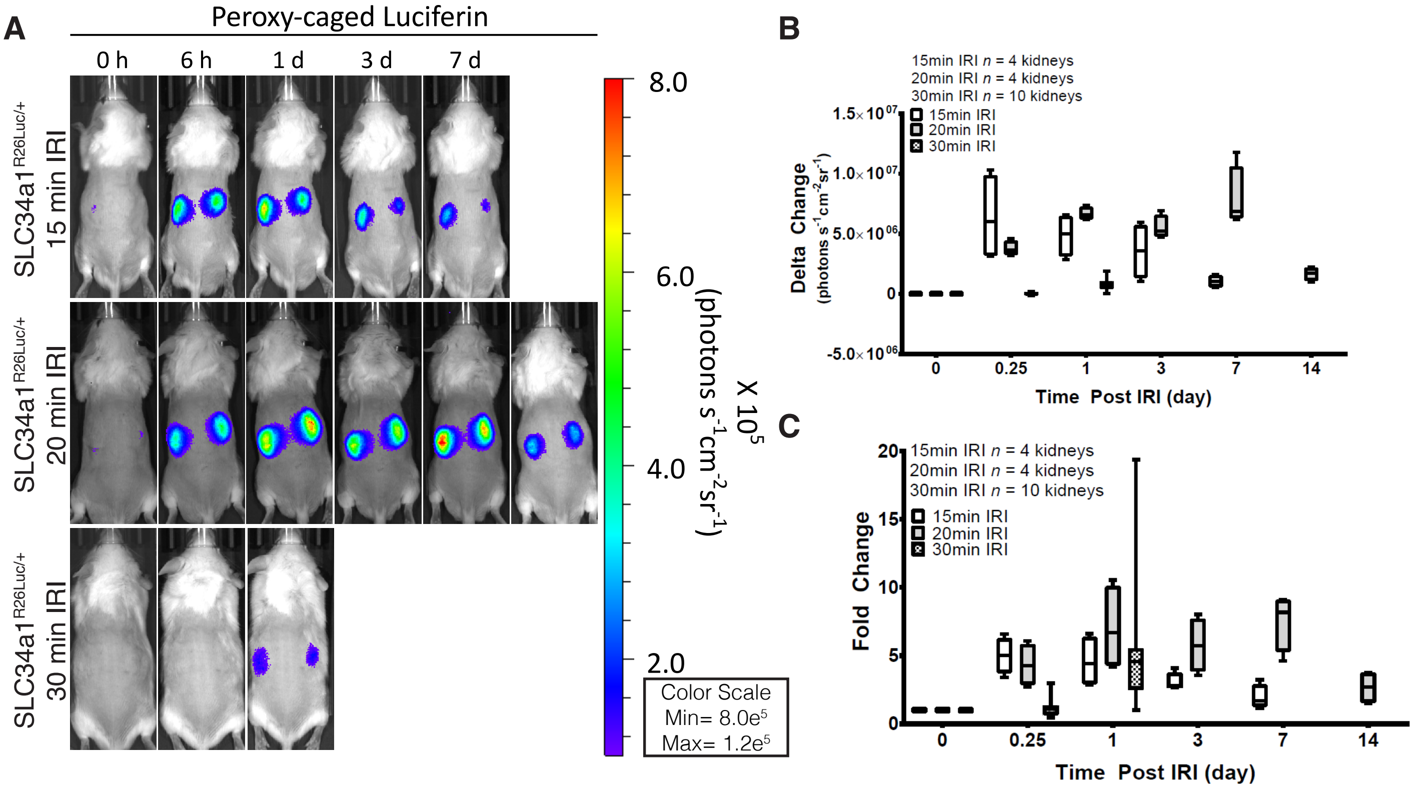


**Supplementary Figure 9. Dose-response and Longitudinal Survival Study of Proximal Tubule H2O2 generation. a** Proximal tubule *Slc34a1*-luciferase reporter (*Slc34a1*$^{R26Luc/+}$) was challenged with 15, 20 and 30 min of IRI and monitored for $H_2O_2$ generation for 7 and 14 days. Experiments were stopped when $H_2O_2$ levels returned to baseline in the 15 and 20 min IRI conditions. **b,c** The highest dose of IRI, 30 min, resulted in lowest expression of the $H_2O_2$ reporter in the proximal tubule.

**Supplementary Table 1**

| Cre recombinase | Segment/Cell-type | Creator |
|---|---|---|
| Podocin-Cre | Glomerulus: Glomerular podocytes | Lawrence B Holzman |
| SLC34a1-CreER(T2) | Proximal tubule: segment S1/S2 (Strong) and S3 (Moderate) | Benjamin Humphreys |
| HoxB7-Cre | Collecting Duct: Principal cells, A/B Intercalated cells and non-A/N | Andrew McMahon |
| E2a-Cre | Global: Germ line | Heiner Westphal |

**Supplementary Table 1.** List of Cre-recombinase animal lines, cell-type and creator.

**Supplementary Table 2**

| Characteristic | **Sham** (*n* = 5) | **3 h** (*n* = 4) | **6 h** (*n* = 4) | **12 h** (*n* = 4) | **24 h** (*n* = 3) |
|---|---|---|---|---|---|
| BUN, mg/dL, median (range) | 23 (13-35) | 45.5 (41-51) | 81 (71-94) | 140 (>140) | 140 (>140) |
| Creatinine, mg/dL, median (range) | 0.2 (<0.2) | 0.8 (0.7-0.9) | 0.95 (0.9-1.1) | 1.8 (1.5-1.8) | 2.5 (2.3-2.8) |
| Sodium, mmol/L, median (range) | 146.5 (142-150) | 144 (141-149) | 138.5 (125-140) | 140.5 (140-141) | 138 (137-146) |
| Potassium, mmol/L, median (range) | 6.15 (5.3-6.7) | 7.55 (6.7-8.2) | 7.4 (5.9-9) | 8.4 (8.3-8.5) | 8.7 (8.6-8.7) |
| Chloride, mmol/L, median (range) | 116.5 (113-123) | 113 (113) | 116 (115-118) | 117 (114-118) | 114 (110-120) |
| TCO2, mmol/L, median (range) | 20.5 (20-22) | 23 (21-24) | 15.5 (9-18) | 13.5 (13-14) | 15 (14-16) |
| Glucose, mg/dL, median (range) | 185.5 (124-326) | 152 (118-212) | 103.5 (84-150) | 89.5 (66-100) | 78 (75-90) |
| Hematocrit, %PCV, median (range) | 38.5 (23-49) | 37 (35-47) | 36.5 (30-41) | 32 (31-32) | 33 (33-34) |
| pH, median (range) | 7.162 (7.151-7.206) | 7.073 (7.053-7.128) | 7.118 (6.932-7.124) | 6.989 (6.965-7.033) | 7.058 (7.013-7.059) |
| PCO2, mmHg, median (range) | 53.2 (46.8-59.1) | 73.55 (57.7-75.9) | 43.75 (38.1-51.3) | 49.9 (44.1-52.5) | 51.1 (43.8-54.2) |
| HCO3, mmol/L, median (range) | 18.9 (18.3-20.7) | 21.15 (19.1-21.6) | 14.25 (8-16.6) | 11.95 (11.8-12) | 13.8 (12.4-14.4) |
| Beecf, mmol/L, median (range) | -9.5 (-10 to -8) | -9 (-10 to -8) | -15 (-24 to -13) | -19.5 (-20 to -19) | -17 (-18 to -16) |
| Anion Gap, mmol/L, median (range) | 17 (6-21) | 18 (15-23) | 15 (14-17) | 20 (19-23) | 21 (18-23) |
| Hemoglobin, g/dL, median (range) | 13.1 (7.8-16.7) | 12.55 (11.9-16) | 12.4 (10.2-13.9) | 10.9 (10.5-10.9) | 11.2 (11.2-11.6) |

**Supplementary Table 2.** Longitudinal blood parameters of IRI animals

**Supplementary Table 3**

| **Assay** | **Sham** (*n* = 8) | **3 h** (*n* = 8) | **6 h** (*n* = 8) | **12 h** (*n*= 8) | **24 h** (*n* = 8) |
|---|---|---|---|---|---|
| MDA, mM, median (range) | 13.97 (9.36-18.20) | 16.84 (9.21-24.45) | 27.3 (19.5-32.98) | 17.98 (12.21-23.63) | 15.08 (11.3-19.63) |
| Protein, mg/ml, median (range) | 24.1 (18-30) | 21.8 (19.7-22.6) | 24.8 (18.6-27.4) | 20.2 (18.5-21.4) | 19.4 (19-20.2) |
| MDA/Protein, nmol/mg, median (range) | 0.5674 (0.3898-0.6686) | 0.8006 (0.4562-1.082) | 1.077 (0.7736-1.444) | 0.8736 (0.5985-1.17) | 0.7555 (0.5885-1.002) |
| MDA Fold, median (range) | 1.04 (0.7-1.36) | 1.26 (0.69-1.82) | 2.04 (1.45-2.46) | 1.34 (0.91-1.76) | 1.12 (0.84-1.46) |
| MDA/Protein Fold, median (range) | 1.03 (0.71-1.22) | 1.46 (0.83-1.97) | 1.96 (1.41-2.62) | 1.59 (1.09-2.13) | 1.38 (1.07-1.82) |

**Supplementary Table 3.** Kidney Lipid Peroxidation

**Supplementary Table 4**

| Gene Name | Symbol | **Sham** (*n* = 8) | **3 h** (*n* = 8) | **6 h** (*n* = 8) | **12 h** (*n* = 8) | **24 h** (*n* = 8) |
|---|---|---|---|---|---|---|
| Lipocalin 2 | Lcn2; 24p3; MSFI; NGAL | 1.005 (0.52-2.09) | 2.405 (1.94-5.01) | 38.06 (2.25-73.14) * | 155.6 (117.9-229.4)**** | 45.72 (33.21-55.67)** |
| Hepatitis A virus cellular receptor 1 | Havcr1; Tim1; KIM-1; TIM-1; Timd1 | 1.00 (0.50-2.26) | 0.52 (0.26-1.20) | 2.815 (0.30-7.04) | 7.30 (3.18-11.08)** | 18.68 (12.77-38.05)**** |
| Heme oxygenase 1 | Hmox1; HO1; HO-1; Hmox; Hemox; Hsp32 | 1.00 (0.65-1.54) | 0.755 (0.53-1.29) | 5.195 (1.78-8.66)**** | 2.365 (1.76-3.45) | 2.85 (2.53-5.37)** |
| Interleukin 1 alpha | Il1a | 1.02 (0.25-4.02) | 8.06 (5.31-15.97)**** | 8.145 (2.54-12.68)**** | 2.34 (0.48-5.02) | 3.83 (1.97-5.80) |
| Interleukin 18 | IL18; Igif | 0.995 (0.54-1.85) | 0.755 (0.50-1.52) | 0.455 (0.36-0.62)*** | 0.435 (0.26-0.61)**** | 0.62 (0.36- 0.90)** |
| Uromodulin | Umod; THP | 0.975 (0.63-1.58) | 1.02 (0.82-1.17) | 0.555 (0.36-0.91)*** | 0.655 (0.35-1.04)** | 0.265 (0.18-0.56)**** |
| Clusterin | Clu; Cli; ApoJ; Sgp2; SP-40; Sgp-2; Sugp-2; | 1.005 (0.53-1.90) | 3.41 (2.15-7.00) | 2.095 (1.13-5.02) | 9.40 (3.16-28.25)**** | 2.10 (1.06-3.90) |
| Epidermal growth factor | Egf | 1.00 (0.56-2.05) | 1.55 (1.12-2.24)* | 0.535 (0.20-0.98)** | 0.405 (0.16-0.73)*** | 0.015 (0.01-0.07)**** |
| Glutathione S-transfer-ase alpha 4 | Gsta4; GST5.7 | 1.00 (0.65-1.55) | 2.145 (1.44-3.11)*** | 0.88 (0.45-1.21) | 2.025 (0.76-3.51)** | 0.59 (0.20-1.36) |
| Heat shock protein 1B | Hspa1b; Hsp70; hsp68; Hsp70-1; Hsp70.1 | 0.93 (0.60-1.68) | 49.71 (41.50-87.43)**** | 19.27 (15.97-56.20)**** | 7.345 (6.01-15.32) | 10.81 (3.73-19.49) |
| Secreted phosphoprotein 1 | Spp1; OP; 2AR; Bsp; Eta; Opn; Ric; BNSP; BSPI; Opnl; Apl-1; ETA-1; Spp-1 | 0.94 (0.74-1.35) | 2.02 (1.34-2.96) | 2.07 (0.74-4.35) | 22.24 (6.86-28.30)**** | 7.375 (2.68-11.93)* |
| Vascular endothelial growth factor A | Vegfa; Vpf; Vegf | 1.035 (0.76-1.31) | 1.105 (0.65-1.47) | 0.435 (0.19-0.87)*** | 0.68 (0.32-1.27) | 0.24 (0.11-0.31)**** |
| Hypoxia Inducible Factor 1 Alpha Subunit | Hif1a | 1.125 (0.59-1.57) | 0.745 (0.59-1.83) | 1.435 (0.49-2.72) | 1.28 (0.36-3.22) | 1.16 (0.45-1.96) |

**Supplementary Table 4 cont**

| Gene Name | Symbol | **Sham** (*n* = 8) | **3 h** (*n* = 8) | **6 h** (*n* = 8) | **12 h** (*n* = 8) | **24 h** (*n* = 8) |
|---|---|---|---|---|---|---|
| Tumor necrosis factor | Tnf; DIF; TNFA; TNFSF2; TNL-G1F; TNF-alpha | 1.005 (0.75-1.33) | 4.06 (1.93-9.09)*** | 1.585 (0.9-2.57) | 1.87 (0.87-4.72) | 2.71 (1.14-4.48) |
| MX dynamin like GT-Pase 1 | Mx1; MX; MxA; IFI78; IFI-78K | 0.975 (0.81-1.24) | 2.095 (0.59-3.33)** | 1.4 (0.35-2.02) | 0.805 (0.32-1.81) | 0.155 (0.08-0.23)* |
| Interleukin 1 beta | IL1b; IL-1; IL1F2; IL1-BETA | 0.985 (0.88-1.14) | 5.5 (1.85-14.77)* | 8.08 (4.84-13.5)** | 3.095 (1.36-16.14) | 5.09 (2.71-15.78)* |
| Interleukin 6 | IL6; CDF; HGF; HSF; BSF2; IL-6; BSF-2; IFNB2; IFN-beta-2 | 1.06 (0.56-1.78) | 85.07 (41.72-216.4) | 484.6 (145.3-1170)**** | 60.24 (17.39-157) | 226 (100.7-548.3)* |
| Complement C3 | C3; ASP; C3a; C3b; AHUS5; ARMD9; CPAMD1; HEL-S-62p | 0.985 (0.79-1.48) | 0.645 (0.24-1.57) | 1.09 (0.42-1.51) | 1.285 (0.15-3.2) | 0.335 (0.23-0.55) |
| Complement C1q A chain | C1q | 1.025 (0.82-1.22) | 0.44 (0.18-1.1)* | 0.595 (0.1-1.49) | 0.86 (0.19-1.4) | 0.405 (0.2-0.55)** |
| Interferon induced protein with tetratricopeptide repeats 1 | Ifit1 | 1.135 (0.58-1.38) | 1.45 (0.63-6.11) | 1.33 (1.15-4.72) | 1.13 (0.36-1.49) | 0.355 (0.21-0.5) |

**Supplementary Table 4.** Longitudinal progression of AKI associated injury genes with qPCR. Fold Change, median (range). Two-way ANOVA $p < 0.05$ *; $p < 0.01$**; $p < 0.001$***; and $p < 0.0001$****.

**Supplementary Table 5**

| Gene Name | Symbol | Sham (N = 4) | Sham Rx (N = 4) | IRI (N = 8) | IRI Rx (N = 10) |
|---|---|---|---|---|---|
| Lipocalin 2 | Lcn2; 24p3; MSFI; NGAL | 0.96 (0.68-1.61) | 0.725 (0.39-1.42) | 28.84 (1.71-55.43)$^{**}$ | 6.335 (1.15-16.2)$^{n/s; **}$ |
| Hepatitis A virus cellular receptor 1 | Havcr1; Tim1; KIM-1; TIM-1; Timd1 | 1.05 (0.48-1.87) | 0.44 (0.32-0.52) | 2.95 (0.32-7.39) | 0.445 (0.18-1.86)$^{n/s; **}$ |
| Heme oxygenase 1 | Hmox1; HO1; HO-1; Hmox; Hemox; Hsp32 | 0.975 (0.87-1.23) | 0.565 (0.38-0.62) | 5.935 (2.04-9.89)$^{**}$ | 3.26 (0.44-3.94)$^{n/s; **}$ |
| Interleukin 1 alpha | Il1a | 1.055 (0.71-1.27) | 0.475 (0.06-0.8) | 7.44 (2.32-11.59)$^{**}$ | 4.2 (0.54-7.34)$^{n/s; *}$ |
| Interleukin 18 | IL18; Igif | 0.985 (0.58-2.15) | 0.535 (0.47-0.63)$^{*}$ | 0.785 (0.63-1.07) | 0.3 (0.22-0.55)$^{n/s; *}$ |
| Uromodulin | Umod; THP | 0.94 (0.54-2.33) | 0.78 (0.53-1.06) | 0.955 (0.62-1.55) | 0.555 (0.32-1.65) |
| Clusterin | Clu; Cli; ApoJ; Sgp2; SP-40; Sgp-2; Sugp-2; | 1.115 (0.49-2.04) | 0.685 (0.61-0.86) | 3.795 (2.04-9.1)$^{*}$ | 1.6 (0.72-4.47)$^{n/s; *}$ |
| Epidermal growth factor | Egf | 1.04 (0.82-1.13) | 0.895 (0.6-1.06) | 0.585 (0.22-1.07)$^{*}$ | 0.65 (0.45-1.22) |
| Glutathione S-transferase alpha 4 | Gsta4; GST5.7 | 1.01 (0.54-1.98) | 0.525 (0.4-0.62) | 1.41 (0.72-1.94) | 0.67 (0.36-0.96)$^{n/s; **}$ |
| Heat shock protein 1B | Hspa1b; Hsp70; hsp68; Hsp70-1; Hsp70.1 | 1.015 (0.5-2.53) | 0.48 (0.16-0.77) | 37.62 (31.18-109.7)$^{***}$ | 29.03 (20.43-35.94)$^{n/s; *}$ |
| Secreted phosphoprotein 1 | Spp1; OP; 2AR; Bsp; Eta; Opn; Ric; BNSP; BSPI; Opnl; Apl-1; ETA-1; Spp-1 | 1.055 (0.52-2.13) | 0.485 (0.4-0.64) | 3.715 (1.34-7.83)$^{*}$ | 1.035 (0.55-3.29)$^{n/s; **}$ |
| Vascular endothelial growth factor A | Vegfa; Vpf; Vegf | 1.025 (0.57-1.85) | 0.745 (0.61-0.88) | 0.685 (0.3-1.36) | 0.62 (0.29-0.82) |
| Hypoxia Inducible Factor 1 Alpha Subunit | Hif1a | 0.95 (0.42-3.51) | 0.385 (0.27-0.93) | 3.21 (1.08-6.06) | 0.5 (0.32-0.9)$^{n/s; ***}$ |
| Tumor necrosis factor | Tnf; DIF; TNFA; TNFSF2; TNLG1F; TNF-alpha | 0.94 (0.58-2.22) | 0.585 (0.23-1.15) | 2.65 (1.5-4.29) | 1.935 (0.88-5.32) |
| MX dynamin like GTPase 1 | Mx1; MX; MxA; IFI78; IFI-78K | 0.91 (0.79-1.54) | 0.63 (0.38-0.72) | 1.74 (0.44-2.51) | 0.94 (0.34-1.62) |

**Supplementary Table 5 cont.**

| Gene Name | Symbol | Sham (N = 4) | Sham Rx (N = 4) | IRI (N = 8) | IRI Rx (N = 10) |
|---|---|---|---|---|---|
| Interleukin 1 beta | IL1b; IL-1; IL1F2; IL1-BETA | 0.99 (0.8-1.27) | 0.915 (0.81-1.15) | 7.135 (4.28-11.93)**** | 4.2 (1.75-7.76)*; ** |
| Interleukin 6 | IL6; CDF; HGF; HSF; BSF2; IL-6; BSF-2; IFNB2; IFN-beta-2 | 1.075 (0.67-1.43) | 0.94 (0.23-2.42) | 342.1 (102.5-826)* | 403.8 (163.7-789.6)** |
| Complement C3 | C3; ASP; C3a; C3b; AHUS5; ARMD9; CPAMD1; HEL-S-62p | 1.07 (0.71-1.24) | 0.84 (0.72-0.92) | 1.16 (0.45-1.61) | 0.665 (0.4-1.41) |
| Complement C1q A chain | C1q | 1.075 (0.6-1.57) | 1.395 (0.39-2.4) | 0.875 (0.15-2.2) | 0.805 (0.38-2) |
| Interferon induced protein with tetratricopeptide repeats 1 | Ifit1 | 0.975 (0.93-1.13) | 16.45 (0.7-2.14) | 1.39 (1.2-4.92) | 1.72 (0.45-7.29) |

**Supplementary Table 5.** qPCR fold change of AKI associated genes after AKI with and without MitoT pre-treatment. Fold Change, median (range). Two-way ANOVA $p < 0.05$ *; $p < 0.01$**; $p < 0.001$***; and $p < 0.0001$****.
