# Supplementary material for: Cell-specific image-guided transcriptomics identifies complex injuries caused by ischemic acute kidney injury in mice
Source: Commun Biol. 2019 Sep 2;2:326. doi: 10.1038/s42003-019-0571-7 (PMC6718519; doi:10.1038/s42003-019-0571-7)
Supplement: Supplementary file 2 — Description of additional supplementary items [file 42003_2019_571_MOESM2_ESM.docx]

**Description of Additional Supplementary Files**

**File Name**: **Supplementary Data 1**

**Description**:   All source data underlying the graphs and charts presented in the main figures

**File Name**: **Supplementary Movie 1**

**Description**:   ***HoxB7^R26Luc/+^* maximum image projection (MIP) movie.** Movie shows a MIP of the reconstructed bioluminescence source inside the animal.  A 20 percent-maximum threshold was used for removing background noise.

**File Name**: **Supplementary Movie 2**

**Description**:   ***Slc34a1^R26Luc/+^* maximum image projection (MIP) movie.** Movie shows a MIP of the reconstructed bioluminescence source inside the animal.  A 20 percent-maximum threshold was used for removing background noise.

**File Name**: **Supplementary Movie 3**

**Description**:   ***Pod^R26Luc/+^* maximum image projection (MIP) movie.** Movie shows a MIP of the reconstructed bioluminescence source inside the animal.  A 20 percent-maximum threshold was used for removing background noise.
