# Supplementary material for: Cell-specific image-guided transcriptomics identifies complex injuries caused by ischemic acute kidney injury in mice
Source: Commun Biol. 2019 Sep 2;2:326. doi: 10.1038/s42003-019-0571-7 (PMC6718519; doi:10.1038/s42003-019-0571-7)
Supplement: Supplementary file 7 — Reporting Summary [file 42003_2019_571_MOESM7_ESM.pdf]

nature research

Corresponding author(s): Neal Paragas

Last updated by author(s): Jul 2, 2019

Reporting Summary

Nature Research wishes to improve the reproducibility of the work that we publish. This form provides structure for consistency and transparency in reporting. For further information on Nature Research policies, see Authors & Referees and the Editorial Policy Checklist.

Statistics

For all statistical analyses, confirm that the following items are present in the figure legend, table legend, main text, or Methods section.

n/a	Confirmed	
☐	☒	The exact sample size (*n*) for each experimental group/condition, given as a discrete number and unit of measurement
☐	☒	A statement on whether measurements were taken from distinct samples or whether the same sample was measured repeatedly
☐	☒	The statistical test(s) used AND whether they are one- or two-sided<br>*Only common tests should be described solely by name; describe more complex techniques in the Methods section.*
☐	☒	A description of all covariates tested
☐	☒	A description of any assumptions or corrections, such as tests of normality and adjustment for multiple comparisons
☐	☒	A full description of the statistical parameters including central tendency (e.g. means) or other basic estimates (e.g. regression coefficient) AND variation (e.g. standard deviation) or associated estimates of uncertainty (e.g. confidence intervals)
☐	☒	For null hypothesis testing, the test statistic (e.g. *F*, *t*, *r*) with confidence intervals, effect sizes, degrees of freedom and *P* value noted<br>*Give P values as exact values whenever suitable.*
☒	☐	For Bayesian analysis, information on the choice of priors and Markov chain Monte Carlo settings
☒	☐	For hierarchical and complex designs, identification of the appropriate level for tests and full reporting of outcomes
☒	☐	Estimates of effect sizes (e.g. Cohen's *d*, Pearson's *r*), indicating how they were calculated

Our web collection on statistics for biologists contains articles on many of the points above.

Software and code

Policy information about availability of computer code

Data collection	LivingImage 4.5 and InVivoAX V1 are commercially available.
Data analysis	LivingImage 4.5, InVivoAX V1, Prism 8, and Excel 16 are commercially available.

For manuscripts utilizing custom algorithms or software that are central to the research but not yet described in published literature, software must be made available to editors/reviewers. We strongly encourage code deposition in a community repository (e.g. GitHub). See the Nature Research guidelines for submitting code & software for further information.

Data

Policy information about availability of data

All manuscripts must include a data availability statement. This statement should provide the following information, where applicable:

- Accession codes, unique identifiers, or web links for publicly available datasets
- A list of figures that have associated raw data
- A description of any restrictions on data availability

Provide your data availability statement here.

Field-specific reporting

Please select the one below that is the best fit for your research. If you are not sure, read the appropriate sections before making your selection.

☒ Life sciences ☐ Behavioural & social sciences ☐ Ecological, evolutionary & environmental sciences

For a reference copy of the document with all sections, see nature.com/documents/nr-reporting-summary-flat.pdf

Life sciences study design

All studies must disclose on these points even when the disclosure is negative.

Sample size	Sample size was determined by using a significant change in serum creatinine, a classic indicator of kidney injury, because using peroxy-caged luciferin as a non-invasive biomarker for oxidative stress in the kidney had not been done previously. Initial power calculation suggested n=6 for a change in serum creatinine. In vivo studies using the caged luciferin revealed that n=4 could power our work using the longitudinal in vivo model.
Data exclusions	No data excluded
Replication	In vivo imaging experiments were conducted longitudinally and in multiple cohorts. Thus reproducibility is inherently show with each N as it is tracked overtime.
Randomization	Control organisms were either the same mouse at the pre-treatment time-point
Blinding	Data was interpreted in a blinded fashion with just the unique identifier known during collection and analysis.

Reporting for specific materials, systems and methods

We require information from authors about some types of materials, experimental systems and methods used in many studies. Here, indicate whether each material, system or method listed is relevant to your study. If you are not sure if a list item applies to your research, read the appropriate section before selecting a response.

Materials & experimental systems

n/a	Involved in the study
☐	☒ Antibodies
☒	☐ Eukaryotic cell lines
☒	☐ Palaeontology
☐	☒ Animals and other organisms
☒	☐ Human research participants
☒	☐ Clinical data

Methods

n/a	Involved in the study
☒	☐ ChIP-seq
☒	☐ Flow cytometry
☒	☐ MRI-based neuroimaging

Antibodies

Antibodies used	AQP2 (C-17) C-terminus of AQP2 of human origin, Goat (Santa Cruz, sc-9882), and mCherry Hig tagged mCherry purified from E. coli Rabbit,(LSBio,LS-C204826)
Validation	Validated in Paragas et al Nature Medicine 2011.

Animals and other organisms

Policy information about studies involving animals; ARRIVE guidelines recommended for reporting animal research

Laboratory animals	ROSA26 L-S-L-Luc/+ [FVB.129S6(B6)-Gt(ROSA)26Sortm1(Luc)Kael/J, 005125] mice were bred with Aqp2-Cre [B6.Cg-Tg(Aqp2-cre)1Dek/J, 006881], EIIa-Cre [B6.FVB-Tg(EIIa-cre)C5379Lmgd/J, 003724], HoxB7-Cre [Tg(Hoxb7-cre)13Amc/J, 004692], Podocin-cre [B6.Cg-Tg(NPHS2-cre)295Lbh/J, 008205] obtained from Jackson Labs, Lcn2-Luciferase reporter from J.M.B, Slc34a1-CreER(T2) from A.K. and Gt(ROSA)26Sor-mCherry-Rpl10a from EMMA.
Wild animals	Study did not involve wild animals
Field-collected samples	Study did not involve samples collected from the field.
Ethics oversight	All animal studies were approved by and performed according to the guidelines approved by the Institutional Animal Care and Use Committee (IACUC) at University of Washington.

Note that full information on the approval of the study protocol must also be provided in the manuscript.
